# Functional analyses of phosphatidylserine/PI(4)P exchangers with diverse lipid species and membrane contexts reveal unanticipated rules on lipid transfer

**DOI:** 10.1186/s12915-021-01183-1

**Published:** 2021-11-20

**Authors:** Souade Ikhlef, Nicolas-Frédéric Lipp, Vanessa Delfosse, Nicolas Fuggetta, William Bourguet, Maud Magdeleine, Guillaume Drin

**Affiliations:** 1 Université Côte d’Azur, Centre National de la Recherche Scientifique, Institut de Pharmacologie Moléculaire et Cellulaire, 660 route des lucioles, 06560 Valbonne, France; 2 Centre de Biologie Structurale, INSERM, CNRS, Université de Montpellier, Montpellier, France; 3 Current position: Department of Chemistry and Biochemistry, University of California San Diego, La Jolla, CA USA

**Keywords:** Lipid transport, Phosphatidylserine, Phosphoinositide, Plasma membrane, Kinetics, Fluorescence

## Abstract

**Background:**

Lipid species are accurately distributed in the eukaryotic cell so that organelle and plasma membranes have an adequate lipid composition to support numerous cellular functions. In the plasma membrane, a precise regulation of the level of lipids such as phosphatidylserine, PI(4)P, and PI(4,5)P_2_, is critical for maintaining the signaling competence of the cell. Several lipid transfer proteins of the ORP/Osh family contribute to this fine-tuning by delivering PS, synthesized in the endoplasmic reticulum, to the plasma membrane in exchange for PI(4)P. To get insights into the role of these PS/PI(4)P exchangers in regulating plasma membrane features, we question how they selectively recognize and transfer lipid ligands with different acyl chains, whether these proteins exchange PS exclusively for PI(4)P or additionally for PI(4,5)P_2_, and how sterol abundance in the plasma membrane impacts their activity.

**Results:**

We measured in vitro how the yeast Osh6p and human ORP8 transported PS and PI(4)P subspecies of diverse length and unsaturation degree between membranes by fluorescence-based assays. We established that the exchange activity of Osh6p and ORP8 strongly depends on whether these ligands are saturated or not, and is high with representative cellular PS and PI(4)P subspecies. Unexpectedly, we found that the speed at which these proteins individually transfer lipid ligands between membranes is inversely related to their affinity for them and that high-affinity ligands must be exchanged to be transferred more rapidly. Next we determined that Osh6p and ORP8 cannot use PI(4,5)P_2_ for exchange processes, because it is a low-affinity ligand, and do not transfer more PS into sterol-rich membranes.

**Conclusions:**

Our study provides new insights into PS/PI(4)P exchangers by indicating the degree to which they can regulate the acyl chain composition of the PM, and how they control PM phosphoinositide levels. Moreover, we establish general rules on how the activity of lipid transfer proteins relates to their affinity for ligands.

**Supplementary Information:**

The online version contains supplementary material available at 10.1186/s12915-021-01183-1.

## Background

Lipid transfer proteins (LTPs) are cytosolic proteins that distribute diverse lipids between organelles, and along with metabolic pathways, regulate the features of cell membranes [1–10]. Some members of a major family of LTPs, the oxysterol-binding protein-related proteins (ORP)/oxysterol-binding homology (Osh) family, vectorially transfer lipids by exchange mechanisms [11]. In yeast, Osh6p and its closest homolog Osh7p transfer phosphatidylserine (PS), an anionic lipid made in the endoplasmic reticulum (ER), to the plasma membrane (PM) [12, 13], where this lipid must be abundant to support signaling pathways. Crystallographic data have revealed that Osh6p consists of one domain—called ORD (OSBP-related domain)—with a pocket that could alternately host one molecule of PS or PI(4)P, a lipid belonging to the class of polyphosphoinositides (PIPs) [12, 13]. The pocket is closed by a molecular lid once the lipid is loaded. These structural data along with in vitro analyses and cellular observations have revealed the following mechanism: Osh6/7p extract PS from the ER and exchange it for PI(4)P at the PM; then they deliver PI(4)P into the ER and take PS once again. This PS/PI(4)P exchange cycle is propelled by the synthesis of PI(4)P from phosphatidylinositol (PI) in the PM and its hydrolysis in the ER membrane, which maintains a PI(4)P concentration gradient between the two compartments.

The PS/PI(4)P exchange mechanism is evolutionarily conserved [14]. Human cells express ORP5 and ORP8 that tether the ER membrane to the PM and exchange PS and PI(4)P between these membranes. They include an N-terminal pleckstrin homology (PH) domain, an ORD resembling Osh6p, and a C-terminal transmembrane segment [15, 16]. They are anchored to the ER by this segment and associate with the PM via their PH domain that targets PI(4)P but also PI(4,5)P_2_ [17–19], which is another essential PIP of the PM [20, 21]. Recently, a complex relationship has been unveiled between the ORP/Osh-mediated PS transfer process and the PI(4,5)P_2_-dependent signaling competences of the cell [17, 19, 22]. It lies in the fact that PI(4)P is both used as a precursor for PI(4,5)P_2_ production and a fuel for PS/PI(4)P exchange. Moreover, as PI(4,5)P_2_, like PI(4)P, serves as anchoring point at the PM for ORP5/8, its level in the PM controls the recruitment and therefore the exchange activity of these LTPs [14, 19]. Consequently, PS/PI(4)P exchange allows for a reciprocal control of PS delivery and PI(4,5)P_2_ synthesis in the PM [19, 22].

Several functional aspects of PS/PI(4)P exchangers remain enigmatic. First, it is unclear whether they selectively transfer certain PS and PI(4)P subspecies at the ER/PM interface. Eukaryotic cells contain a repertoire of PS and PIP subspecies with acyl chains of different lengths and unsaturation degrees. The nature and proportion of each subspecies in these repertoires vary considerably between organisms (e.g., yeasts and mammals [23–25]) but also cell types and tissues in mammals [26, 27]. Moreover, inside the cell, the relative proportion of each PI(4)P and PS subspecies differs among organelles [28, 29]. Some of these subspecies are predominant and this might have functional reasons. For instance, certain unsaturated PS species seem to associate preferentially with sterol in the PM, which could control the transversal distribution of sterol and the lateral distribution of PS [30, 31], and thereby the asymmetry and remodeling propensity of this membrane. Also functional lipid nanodomains are formed by the association of 18:0/18:1-PS with very-long-chain sphingolipids [25]. A hallmark of mammal cells is the dominance of polyunsaturated PIPs with 18:0/20:4 acyl chains. This seems critical for the maintenance of a PI(4,5)P_2_ pool and of PI(4,5)P_2_-dependent signaling processes in the PM via the so-called PI cycle [32, 33]. Consequently, it is worth analyzing how precisely ORP/Osh proteins transfer PS and PI(4)P species with different acyl chains, in order to define how selective they can be and to what extent they can contribute to the tuning of lipid homeostasis in the PM.

A second issue concerns the links between the ORP/Osh-mediated PS transfer process and the regulation of PI(4,5)P_2_ levels. It has been reported recently that ORP5/8 use PI(4,5)P_2_ rather than PI(4)P as a counterligand for supplying the PM with PS [17]. This would mean that the PI(4,5)P_2_ level in the PM is directly lowered by the consumption of PI(4,5)P_2_ during exchange cycles. Yet, this conclusion is disputed [19] and remains surprising in view of the very first structural analyses that suggest that the polar head of PI(4,5)P_2_, unlike that of PI(4)P, cannot be accommodated by the ORD due to steric constraints [34]. The structures of the ORD of ORP1 and ORP2 in complex with PI(4,5)P_2_ have been solved [35, 36] but they revealed that the PI(4,5)P_2_ molecule is only partially buried in the binding pocket. All these observations raise doubts about the existence of functional PI(4,5)P_2_-bound forms of ORPs, including ORP5 and ORP8, in the cell.

Third, as mentioned above, unsaturated PS and sterol preferentially associate with each other in the PM [30, 31]. In parallel, it has been recently proposed that in the yeast PM, unsaturated PS and PI(4)P co-distribute in the presence of sterol to synergistically stimulate PI(4)P 5-kinase activity and promote a robust PI(4)P-to-PI(4,5)P_2_ conversion [22]. Osh proteins, such as Osh6/7p but also Osh4/5p which are sterol/PI(4)P exchangers, control the formation of these domains. Considering all these observations, one might wonder whether the tight association of sterol with PS acts as a thermodynamic trap that aids PS/PI(4)P exchangers to accumulate PS in the PM and thus contribute to the coupling between PS transfer and PI(4,5)P_2_ synthesis.

Here, we addressed these three interrelated questions by conducting in vitro functional analyses of Osh6p and ORP8, combined with simulations and cellular observations. Using a large set of PS subspecies, we found that these LTPs transfer unsaturated PS more slowly than saturated PS between liposomes. In contrast, in a situation of PS/PI(4)P exchange, only the transfer of unsaturated PS species is largely accelerated, and efficient exchange occurs with certain unsaturated PS and PI(4)P species that are prominent in cells. Unexpectedly, by measuring the affinity of Osh6p and ORP8 for PS and PI(4)P subspecies and correlating these data with transfer rates, we established that the simple transfer of high-affinity ligands is slower than that of low-affinity ligands. Next we found that high-affinity ligands are rapidly transferred only if they can be exchanged for ligands of equivalent affinity. Furthermore, we determined that, if PI(4)P and PI(4,5)P_2_ are both accessible to Osh6p and ORP8, PI(4,5)P_2_ cannot be transferred or exchanged for PS because PI(4,5)P_2_ is a low-affinity ligand. This suggests that PI(4,5)P_2_ cannot be transported by ORP/Osh proteins in cells. Finally, we found that the activity of PS/PI(4)P exchangers barely changes on sterol-rich membranes. Our study provides insights into PS/PI(4)P exchangers but also sets general rules on how the activity of LTPs relates to their affinity for lipids, which improves our knowledge of lipid transfer.

## Results

### Osh6p and ORP8 transfer saturated and unsaturated PS species differently

**Fig. 1:**
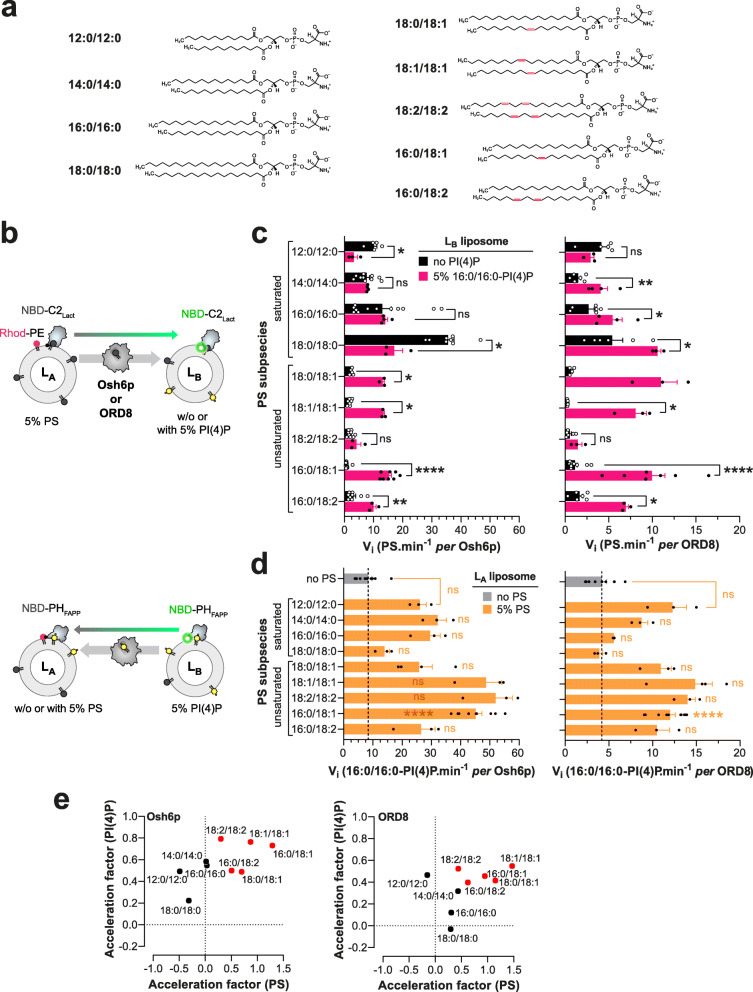
PS transfer and PS/PI(4)P exchange activity of Osh6p and ORD8 measured with different PS subspecies. **a** Name and chemical structure of the different PS subspecies. **b** Description of FRET-based protocols to measure PS transfer from *L*_A_ to *L*_B_ liposomes using the NBD-C2_Lact_ sensor, and PI(4)P transfer along the opposite direction using NBD-PH_FAPP_. **c** Initial transfer rates determined for each PS subspecies. Osh6p (200 nM) or ORD8 (240 nM) was added to *L*_A_ liposomes (200 μM total lipid), made of DOPC and containing a given PS species at 5%, mixed with *L*_B_ liposomes (200 μM) containing or not 5% 16:0/16:0-PI(4)P and 250 nM NBD-C2_Lact_. Data are represented as mean ± s.e.m. (Osh6p, non-exchange condition, *n* = 3–16; Osh6p, exchange condition, *n* = 3–8; ORD8, non-exchange condition, *n* = 3–11; ORD8_,_ exchange condition, *n* = 3–7). Statistically significant differences between PS transfer rates measured under non-exchange and exchange conditions were determined using an unpaired Mann–Whitney *U* test; **** *p* < 0.0001, **p < 0.01, *p < 0.05, ns: not significant. **d** Initial PI(4)P transfer rate. *L*_B_ liposomes containing 5% 16:0/16:0-PI(4)P were mixed with *L*_A_ liposomes, containing or not a given PS species (at 5%), and with Osh6p (200 nM) or ORD8 (240 nM) in the presence of 250 nM NBD-PH_FAPP_. Data are represented as mean ± s.e.m. (Osh6p, non-exchange condition, *n* = 9; Osh6p, exchange condition, *n* = 3–9; ORD8, non-exchange condition, *n* = 9; ORD8_,_ exchange condition, *n* = 3–10). An unpaired Mann–Whitney *U* test was used to determine the statistically significant differences between the PI(4)P transfer rates measured in non-exchange and exchange conditions; ****p < 0.0001 , **p < 0.01, *p < 0.05, ns: not significant. **e** Acceleration of PS transfer as a function of the acceleration of PI(4)P transfer determined from rates measured in non-exchange and exchange conditions with all PS subspecies

We first measured in vitro the speed at which Osh6p and the ORD of ORP8 (ORP8 [370-809] [14], hereafter called ORD8) transferred PS subspecies with different acyl chains between two membranes (Fig. 1a). Our series comprised subspecies with saturated acyl chains of increasing length (12:0/12:0, 14:0/14:0, 16:0/16:0, 18:0/18:0), with two C18 acyl chains that are more or less unsaturated (18:0/18:1, 18:1/18:1, 18:2/18:2) and with one saturated C16 acyl chain at the sn-1 position and one C18 acyl chain, with a different unsaturation degree, at the sn-2 position (16:0/18:1, 16:0/18:2). Note that 16:0/18:1-PS is the dominant PS species in *S. cerevisiae* yeast [22, 24, 28, 37] (under standard growing conditions), whereas in humans, 18:0/18:1-PS and 16:0/18:1-PS are the two most abundant species [25, 38–40]. PS transfer was measured between *L*_A_ liposomes, made of DOPC and containing 5% PS (mol/mol) and 2% Rhod-PE, and *L*_B_ liposomes only made of DOPC, using the fluorescent sensor NBD-C2_Lact_. In each measurement, NBD-C2_Lact_ was initially bound to *L*_A_ liposomes and its fluorescence was quenched due to energy transfer to Rhod-PE; when LTP transferred PS to *L*_B_ liposomes, NBD-C2_Lact_ translocated onto these liposomes and the fluorescence increased (Fig. 1b). By normalizing the NBD signal, we established transfer kinetics (Additional file 1: Fig. S1a) and initial transfer rates (Fig. 1c). Osh6p transferred saturated PS rapidly, with rates between 7.6 ± 1 (mean ± s.e.m., 14:0/14:0-PS) and 35.2 ± 2 PS min^−1^ (18:0/18:0-PS). The transfer of unsaturated PS species was much slower (from 0.8 ± 0.1 to 3.1 ± 0.8 PS min^−1^ *per* Osh6p). A different picture was obtained when we measured PS transfer in a situation of PS/PI(4)P exchange using *L*_B_ liposomes containing 5% 16:0/16:0-PI(4)P. The transfer rates measured with 14:0/14:0-PS and 16:0/16:0-PS were similar to those measured in non-exchange contexts, and significantly lower with 12:0/12:0-PS and 18:0/18:0-PS. In contrast, the transfer rate of unsaturated PS, with the exception of 18:2/18:2-PS, strongly increased (by a factor from 3.2 to 19.5).

Overall, ORD8 transported PS more slowly than Osh6p in both exchange and non-exchange situations (Fig. 1c, Additional file 1: Fig. S1b). Nevertheless, the activity of ORD8 changed depending on the PS subspecies in a manner similar to that of Osh6p, as highlighted by the correlation of transfer rates measured for the two LTPs with each PS ligand (*R*^2^ ~ 0.75, Additional file 1: Fig. S1c). Like Osh6p, ORD8 transferred saturated PS species more rapidly than unsaturated ones when PI(4)P was absent. In a situation of PS/PI(4)P exchange, the transfer of unsaturated PS species (except for 18:2/18:2-PS) was much more rapid (up to 29-fold) whereas the transfer of saturated PS was slightly enhanced or inhibited. Collectively, these data did not point to a monotonic relationship between PS transfer rates and the length of PS acyl chains or the degree of unsaturation of these chains. However, they indicated that PS species were transported and exchanged with PI(4)P quite differently depending on whether or not they had at least one double bond.

### Coupling between the transfer rate of PS species and PI(4)P under exchange conditions

Next, we determined whether the rate of 16:0/16:0-PI(4)P transfer was different depending on the nature of the PS species under the exchange conditions. Using a fluorescent PI(4)P sensor (NBD-PH_FAPP_) and a FRET-based strategy akin to that used to measure PS transfer, we measured the speed at which PI(4)P, at 5% in *L*_B_ liposomes, was transported by Osh6p and ORD8 to *L*_A_ liposomes devoid of PS or containing a given PS species (at 5%) (Fig. 1b, Additional file 1: Fig. S2). With PS-free *L*_A_ liposomes, the initial PI(4)P transfer rate was 8.4 ± 1.3 PI(4)P min^−1^ for Osh6p and 4.2 ± 0.5 PI(4)P min^−1^ for ORD8 (Fig. 1d). In a situation of lipid exchange, these transfer rates increased to a different degree when *L*_A_ liposomes contained a PS species other than 18:0/18:0-PS and, in experiments with ORD8, 16:0/16:0-PS (Fig. 1d). For each PS species, we calculated an acceleration factor corresponding to the ratio (expressed as a log value) of the PI(4)P transfer rate, measured in the presence of this PS species, to the PI(4)P transfer rate measured in the absence of counterligand. Also, acceleration factors based on PS transfer rates reported in Fig. 1c were determined. Then for each LTP, these two factors were plotted against each other, allowing saturated and unsaturated PS species to cluster in two groups (Fig. 1e). With Osh6p, the group including saturated PS species was characterized by null or negative acceleration factors for PS (down to − 0.49) associated with low or moderate acceleration factors for 16:0/16:0-PI(4)P (from 0.20 to 0.58). In contrast, the group corresponding to unsaturated PS species was characterized by higher acceleration factors for both PS (from 0.30 to 1.30) and PI(4)P (from 0.49 to 0.79). With ORD8, the acceleration factors for saturated PS were negative, null, or moderate (from − 0.15 to 0.47) and associated with null or moderate acceleration factors for PI(4)P (from 0 to 0.48). For unsaturated PS, the acceleration factors were higher, ranging from 0.44 to 1.47 for PS and from 0.40 to 0.55 for PI(4)P. The observation of high acceleration factors for both unsaturated PS and 16:0/16:0-PI(4)P, and much lower or even negative values for saturated PS species, suggests that the LTPs exchange unsaturated PS for PI(4)P much more efficiently than saturated PS.

### Exchange activity with prominent cellular PS and PI(4)P species

We next measured how Osh6p and ORD8 exchanged PS and PI(4)P species that are dominant in the yeast and/or human repertoire. With Osh6p, we tested 16:0/18:1-PS and 18:0/18:1-PS with 16:0/18:1-PI(4)P, one of the two most abundant yeast PI(4)P species [22, 23]. As a comparison, we tested a non-yeast species, 18:0/20:4-PI(4)P, which is the main constituent of purified brain PI(4)P. With ORD8, we tested the two same PS species with 18:1/18:1-PI(4)P that resembles unsaturated PI(4)P species (36:1 and 36:2) found in transformed cells [41, 42] and 18:0/20:4-PI(4)P, which is prominent in primary cells and tissues [42]. As a comparison, we used 16:0/16:0-PI(4)P as in our previous assays.

**Fig. 2:**
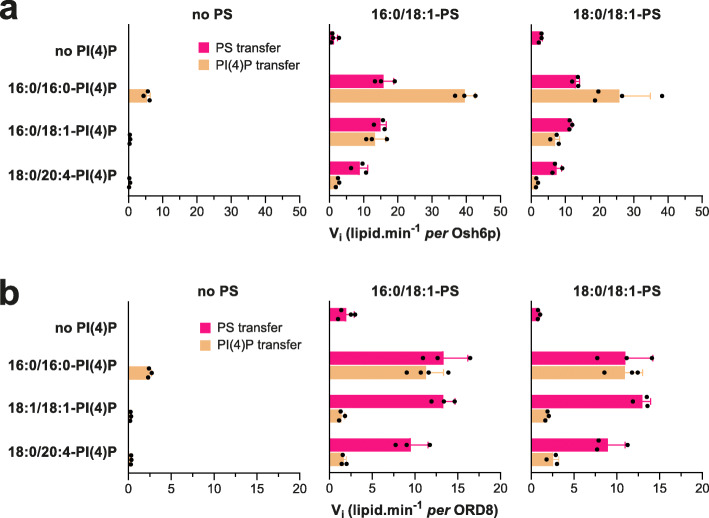
Ability of Osh6p and ORP8 to transfer and exchange cellular PS and PI(4)P species. **a** Initial PS and PI(4)P transfer rates, measured along opposite directions, between *L*_A_ and *L*_B_ liposomes (200 μM total lipid each) with Osh6p (200 nM) at 30 °C, in the absence of counterligand or in a situation of lipid exchange, with diverse combinations of PS (16:0/18:1 or 18:0/18:1, 5% in *L*_A_ liposomes) and PI(4)P species (16:0/16:0, 16:0/18:1 or 18:0/20:4, 5% in *L*_B_ liposomes). Data are represented as mean ± s.e.m. (*n* = 3–4). **b** Similar experiments were conducted with ORD8 (240 nM) at 37 °C using 18:1/18:1-PI(4)P instead of 16:0/18:1-PI(4)P. Data are represented as mean ± s.e.m. (*n* = 3–4)

Osh6p slowly transferred the two PS species from *L*_A_ to *L*_B_ liposomes in the absence of PI(4)P but ten times faster when *L*_B_ liposomes contained 16:0/18:1-PI(4)P or 16:0/16:0-PI(4)P. Smaller accelerations of PS transfer were seen with 18:0/20:4-PI(4)P as counterligand. Conversely, in the absence of PS, Osh6p hardly transported any 16:0/18:1-PI(4)P and 18:0/20:4-PI(4)P (< 0.4 lipids min^−1^) compared to saturated PI(4)P (5.4 lipids min^−1^) (Fig. 2a, Additional file 1: Fig. S3a, b). When *L*_A_ liposomes contained PS, the transfer rate of all PI(4)P species increased but with rates that were high for 16:0/16:0-PI(4)P (25.8–39.6 lipids min^−1^), intermediate for 16:0/18:1-PI(4)P (7–13.3 lipids min^−1^) and low for 18:0/20:4-PI(4)P (1.63–2.4 lipids min^−1^). Interestingly, the 16:0/18-1-PS and 16:0/18:1-PI(4)P transfer rates were both similar and high in a situation of lipid exchange, suggesting that Osh6p can execute an efficient one-for-one exchange of these major yeast PS and PI(4)P species.

In the absence of PI(4)P, ORD8 slowly transferred 16:0/18:1-PS and 18:0/18:1-PS between membranes and much faster in a situation of exchange, by a factor of 4.8–6.8 and 11.5–16.6, respectively, depending on the PI(4)P species used as counterligand (Fig. 2b, Additional file 1: Fig. S3c, d). Like Osh6p, ORD8 barely transferred unsaturated PI(4)P under non-exchange conditions (< 0.26 lipids min^−1^), compared to 16:0/16:0-PI(4)P. Under exchange conditions, ORD8 transferred these PI(4)P species more rapidly but far less than PS in the opposite direction. This suggests that ORP8 cannot efficiently exchange unsaturated PS for PI(4)P. We conclude that the acyl chain composition of PI(4)P, and primarily its unsaturation degree, impacts how Osh6p and ORP8 transfer and use this PIP in exchange for PS.

### Osh6p and ORP8 activities drastically change if the sn-1 or sn-2 chain of PS is monounsaturated

**Fig. 3:**
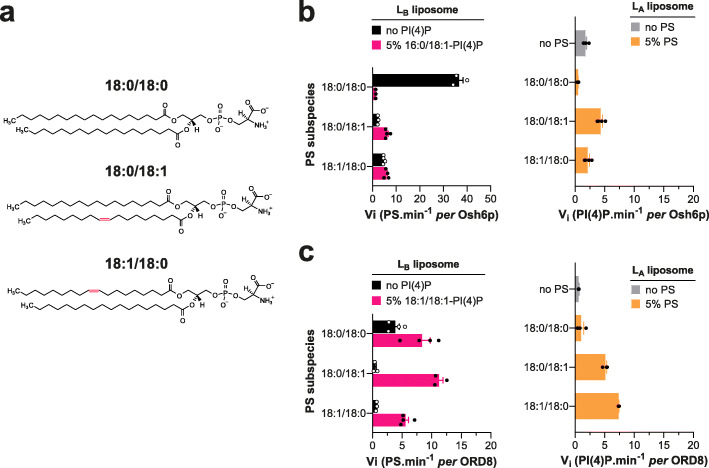
Transfer of 18:1/18:0-PS *vs* 18:0/18:1-PS by Osh6p and ORD8. **a** Chemical structure of 18:1/18:0-PS compared to that of 18:0/18:0-PS and 18:0/18:1-PS. **b** Initial rates of PS and PI(4)P transfer, measured along opposite directions, between *L*_A_ and *L*_B_ liposomes (200 μM total lipid each) with Osh6p (200 nM) at 30 °C, in non-exchange or exchange contexts with 18:0/18:0-PS, 18:0/18:1-PS, or 18:1/18:0-PS (5% in *L*_A_ liposomes) and 16:0/18:1-PI(4)P (5% in *L*_B_ liposomes). Data are represented as mean ± s.e.m. (*n* = 3–4). **c** Similar experiments were performed with ORD8 (240 nM) at 37 °C using 18:1/18:1-PI(4)P instead of 16:0/18:1-PI(4)P

Striking differences were seen between 18:0/18:0-PS and unsaturated forms of this lipid in our transfer assays. In particular, data obtained with 18:0/18:0-PS and 18:0/18:1 PS suggested that only one double bond in PS was sufficient to drastically change LTP activity. Whether this depends on the location of this double bond in the sn-2 chain was unclear. Therefore, we compared how Osh6p transferred 18:0/18:1 PS and 18:1/18:0-PS, in which the saturated and monounsaturated acyl chains are permuted, between membranes (Fig. 3a). In mere transfer assays, 18:0/18:1-PS and 18:1/18:0-PS were transported at rates that were slightly different (4.7 vs 2.1 PS min^−1^) but ten-fold more slowly than 18:0/18:0-PS (Fig. 3b). In the presence of 16:0/18:1-PI(4)P, the transfer of the two unsaturated PS species was faster whereas the transfer of 18:0/18:0-PS was inhibited. The opposite transfer of 16:0/18:1-PI(4)P was inhibited if 18:0/18:0-PS was tested as counterligand but enhanced using the two unsaturated PS forms (Fig. 3b). Similar results were obtained with ORD8 except that the transfer of 18:0/18:0-PS was slightly more rapid (by 2.2-fold) in exchange conditions (here 18:1/18:1-PI(4)P was used as counterligand (Fig. 3c3c)). However, the rate of acceleration was low compared to that measured with 18:0/18:1-PS and 18:1/18:0-PS (by 24.0 and 8.4-fold, respectively). Jointly, these results indicate that only one double bond, in one or the other acyl chain of PS, is enough to dramatically change how the two LTPs transport and exchange this lipid for PI(4)P.

### Osh6p and ORD8 have a higher affinity for unsaturated than saturated PS and PI(4)P species

**Fig. 4:**
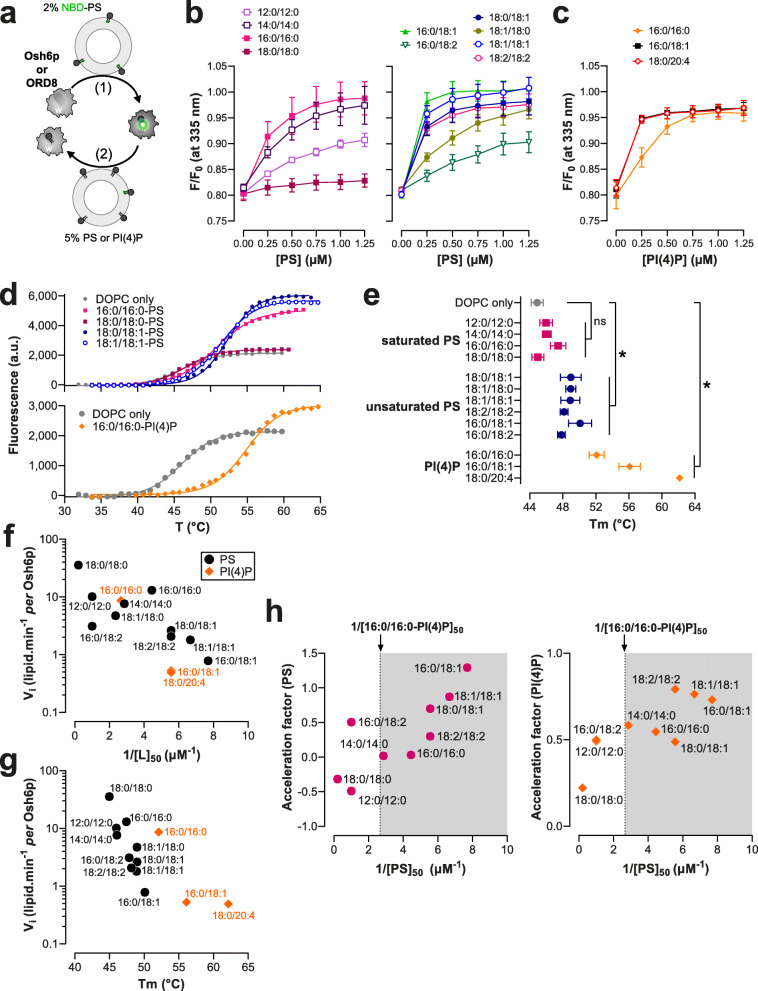
Relationship between the affinity of Osh6p for PS and PI(4)P species and its capacity to transfer them. **a** Principle of the NBD-PS-based competition assays. The tryptophan (W) fluorescence of Osh6p and ORD8 is quenched when they host an NBD-PS molecule. The replacement of NBD-PS by unlabelled PS restores the fluorescence of these proteins. **b** Competition assays with different PS species. Liposomes (100 μM total lipid, final concentration), made of DOPC and doped with 2% NBD-PS, were added to Osh6p (240 nM) in HK buffer at 30 °C. The sample was excited at 280 nm and the emission was measured at 335 nm. Incremental amounts of liposome, containing a given PS species at 5%, were added to the sample. The fluorescence was normalized considering the initial *F*_max_ fluorescence, prior to the addition of NBD-PS-containing liposomes, and the dilution effects due to liposome addition. Data are represented as mean ± s.e.m. (*n* = 3). **c** Competition assays with liposomes containing either 5% 16:0/16:0-PI(4)P, 16:0/18:1-PI(4)P, or 18:0/20:4-PI(4)P. Data are represented as mean ± s.e.m. (*n* = 4 for 16:0/18:1-PI(4)P, *n* = 3 for other PI(4)P species). **d** Melting curves of Osh6p loaded with different PS species or 16:0/16:0-PI(4)P. In a typical measurement, a sample containing 5 μM of protein and 5× SYPRO Orange in HK buffer was heated and fluorescence was measured at *λ*_em_ = 568 nm (*λ*_ex_ = 545 nm). A control experiment with Osh6p incubated with DOPC liposomes devoid of lipid ligands is shown (DOPC only). Only a few curves corresponding to representative ligands are shown for clarity. **e** Melting temperatures (*T*_m_) determined for Osh6p pre-incubated with pure DOPC liposome or loaded with diverse PS and PI(4)P subspecies. Data are represented as mean ± s.e.m (*n* = 3–5). Pairwise comparison by unpaired Mann–Whitney *U* test of *T*_m_ rate measured with Osh6p in apo form (DOPC only) and Osh6p loaded with saturated or unsaturated PS species, or a PI(4)P species; *p < 0.05, ns: not significant. **f** Initial transfer rates determined in non-exchange contexts for PS and PI(4)P subspecies with Osh6p (shown in Figs. 1b,c, 2, and 3) as a function of 1/[L]_50_ values determined for each lipid subspecies. **g** PS transfer rates in non-exchange conditions as a function of *T*_m_ values determined with Osh6p. **h** Acceleration factors determined for PS and PI(4)P from experiments shown in Fig. 1b and c1b and c, as a function of the 1/[L]_50_ values determined for each PS subspecies. The 1/[L]_50_ value determined with 16:0/16:0-PI(4)P is indicated

Our results suggest that Osh6p and ORD8 transport and exchange PS and PI(4)P at a different speed depending on the unsaturation degree of these lipids. To further analyze why, we devised assays to determine the relative affinity of these LTPs for each PS and PI(4)P species. We established that the intrinsic fluorescence of both proteins (from tryptophan, with a maximum intensity at *λ* = 335 and 340 nm, respectively) was quenched by ~ 25% when mixed with liposomes doped with 2% NBD-PS, a PS species whose C12:0 acyl chain at the sn-2 position bears an NBD moiety (Additional file 1: Fig. S4). Concomitantly, a higher NBD fluorescence was measured at *λ* = 540 nm. Adding each LTP to liposomes doped with 2% NBD-PC provoked a slighter decrease of tryptophan fluorescence, yet similar to the changes recorded with pure PC liposomes, and no change in NBD fluorescence. Likely, FRET exclusively occurs between these proteins and NBD-PS because this lipid is specifically trapped in their binding pocket and close to a number of tryptophan residues. Interestingly, we found subsequently that Osh6p and ORP8 fluorescence, pre-mixed with NBD-PS-containing liposomes, increased when adding incremental amounts of liposomes containing unlabelled PS. This allowed us to measure how each PS species competes with NBD-PS for occupation of the Osh6p and ORD8 pocket and thus determine the relative affinity of each ORD for different lipid ligands (Fig. 4a, b, Additional file 1: Fig. S5a and Additional file 2: Table S1 and Table S5). Remarkably, Osh6p had a very low affinity for 18:0/18:0-PS and 12:0/12:0-PS. It showed a higher affinity for 14:0/14:0-PS and 16:0/16:0-PS. Highest affinities were found with unsaturated PS and more particularly 16:0/18:1-PS, 18:1/18-1-PS and 18:0/18:1-PS. Interestingly, Osh6p had a higher affinity for 18:0/18:1-PS than its mirror 18:1/18:0-PS counterpart (Fig. 4b and Additional file 2: Table S1). Using this assay, we also found that Osh6p had a high affinity for 16:0/18:1-PI(4)P and 18:0/20:4-PI(4)P and less for 16:0/16:0-PI(4)P (Fig. 4c, Additional file 2: Table S2). With ORD8, competition assays revealed that it had a much lower affinity for saturated PS than for unsaturated PS (Additional file 1: Fig. S5a, Additional file 2: Table S5). ORD8 had a higher affinity for 18:1/18:1-PI(4)P and 18:0/20:4-PI(4)P than for 16:0/16:0-PI(4)P (Additional file 1: Fig. S5b, Additional file 2: Table S6). Collectively, our data indicated that Osh6p and ORD8 had a higher affinity for unsaturated than saturated lipid ligands.

Alternatively, Osh6p was incubated with liposomes doped with a given PS or PI(4)P subspecies, isolated, and then subjected to thermal shift assays (TSAs) to evaluate to what extent it formed a stable complex with each ligand ((Fig. 4d,e and Additional file 2: Table S3). Low melting temperatures (*T*_m_) were observed with Osh6p exposed to liposomes containing saturated PS species (from 45 to 47.4 °C), near the *T*_m_ value of Osh6p incubated with DOPC liposomes devoid of ligand (44.9 ± 0.7 °C). In contrast, significantly higher values were obtained with unsaturated PS (from 47.8 to 50.1 °C). The highest *T*_m_ values were found with Osh6p loaded with 16:0/18:1 and 18:0/20:4-PI(4)P species (56.1 and 62.1 °C, respectively), and a slightly lower *T*_m_ was found with 16:0/16:0-PI(4)P (52 °C). These results suggest that Osh6p is more prone to capture and hold unsaturated PS and PI(4)P than saturated PS, corroborating the results from competition assays.

### The affinity of Osh6p and ORP8 for PS and PI(4)P species dictates how they transfer and exchange them

Next, we analyzed how the affinity of Osh6p and ORD8 for lipid ligands was related to their capacity to transfer them. To do so, we plotted the PS and PI(4)P transfer rates measured in non-exchange contexts (reported in Fig. 1c, d, Fig. 2, and Fig. 3) as a function of 1/[L]_50_, with [L]_50_ being the concentration of each species necessary to displace 50% NBD-PS from each LTP in the competition assays. Remarkably, this revealed an inverse relationship between the transfer rates and 1/[L]_50_ values (Fig. 4f and Additional file 1: Fig. S5c) for each LTP. For Osh6p, plotting the transfer rates as a function of *T*_m_ values uncovered a comparable relationship (Fig. 4 g). This suggested that the less affinity these LTPs have for a ligand, the faster they transfer it between membranes. We also plotted the acceleration factors established under exchange conditions with different PS species and 16:0/16:0-PI(4)P (showed in Fig. 1e) against 1/[L]_50_ values determined with each PS species and LTP (Fig. 4 h, Additional file 1: Fig. S5d). Interestingly, a positive relationship was found between the 1/[L]_50_ values and each of these factors. Moreover, we noted that PS and PI(4)P transfer rates measured under exchange conditions were overall higher when the LTPs had a higher affinity for PS than PI(4)P (1/[PS]_50_ > 1/[16:0/16:0-PI(4)P]_50_). Collectively, these analyses reveal an inverse correlation between the affinity of an LTP for a ligand and its ability to simply transfer it down its concentration gradient. Moreover, they indicate that in a situation of lipid exchange, the acceleration of the PS and PI(4)P transfer rate is more pronounced with high-affinity PS species.

#### Simulation of transfer and exchange activity of the ORD as a function of its affinity for PS and PI(4)P

**Fig. 5:**
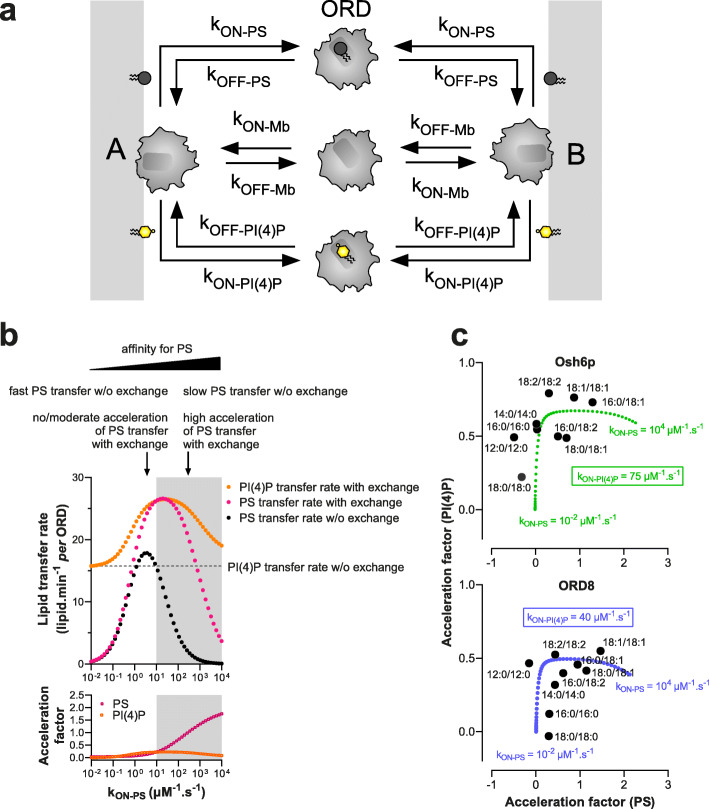
Analysis of the relationship between ORD’s affinity for PS and PI(4)P and its ability to transfer these lipids between membranes. **a** Description of the kinetic model. Osh6p or ORD8 (ORD) interacts with the same affinity with two distinct membranes A and B, each harboring a PS and PI(4)P pool, and can extract and release PS and PI(4)P. ORD-PS and ORD-PI(4)P correspond to the ORD in 1:1 complex with PS and PI(4)P, respectively. All *k*_ON_ and *k*_OFF_ rates were set to 10 μM^−1^ s^−1^, and 1 s^−1^, respectively, unless otherwise specified. **b** Initial PS transfer rate (gray dots) as a function of *k*_ON-PS_ values (ranging from 0.01 to 10,000 μM^−1^ s^−1^), under the condition where the A membrane initially contained 5% PS and B membrane was devoid of PI(4)P (non-exchange condition). Initial PS (pink dots) and PI(4)P transfer rates (orange dots) were also calculated as a function of *k*_ON-PS_, considering that PS and PI(4)P were initially present at 5% in the A and B membranes, respectively (exchange condition). PI(4)P transfer rate simulated with A membrane devoid of PS (non-exchange condition) was indicated by a dashed line. The gray areas correspond to regimes where *k*_ON-PS_ > *k*_ON-PI(4)P_, i.e., the ORD has more affinity for PS than PI(4)P. The acceleration factors, calculated for PS and PI(4)P, correspond to the ratio (in log value) between the transfer rates derived from simulations performed in exchange and non-exchange conditions. **c** Acceleration factors of PS and PI(4)P transfer in exchange conditions, established for Osh6p and ORD8 with different PS species and 16:0/16:0-PI(4)P, are plotted against each other as in the Fig. 1e. For comparison, theoretical acceleration factors of PS and PI(4)P transfer, considering *k*_ON-PS_ value ranging from 0.01 to 10,000 μM^−1^ s^−1^ and a *k*_ON__-__PI(4)P_ value of 75 μM^−1^ s^−1^ for Osh6p or 40 μM^−1^ s^−1^ for ORD8, are plotted against each other (Osh6p, green; ORD8, blue)

To understand why the affinity of Osh6p and ORD8 for PS and PI(4)P species governed how they transferred and exchanged these lipids, we built a simplified kinetic model (Fig. 5a). It was assumed that the ORD interacts similarly with A and B membranes during a transfer process (*k*_ON-Mb_= 10µM^–1^ s^−1^and *k*_OFF-Mb_ = 0.1 s^−1^) with an equal ability to capture and release a given lipid (similar *k*_ON-lipid_ and *k*_OFF-lipid_). We simulated initial PS transfer rates for *k*_ON-PS_ values ranging from 10^−2^ to 10^4^ μM^−1^ s^−1^ to evaluate how the affinity of the ORD for PS (proportional to k_ON-PS_) governs how it transfers this lipid. The *k*_ON-PI4P_ values was set to 10 μM^−1^ s^−1^ and *k*_OFF-PS_ and *k*_OFF-PI4P_ values to 1 s^−1^; the ORD concentration was 200 nM, with an A membrane including 5 μM accessible PS and a B membrane devoid of ligand (as in our transfer assays). A bell-shaped curve (Fig. 5b, black dots) was obtained with a maximum at *k*_ON-PS_ = 3.7 μM^−1^ s^−1^ and minima near zero for very low and high *k*_ON-PS_ values. Remarkably, our simulations indicate that an LTP can transfer a low-affinity ligand more rapidly than a high-affinity one, as seen for instance when comparing rates at *k*_ON-PS_ = 10 and 100 μM^−1^ s^−1^, and as observed experimentally with saturated and unsaturated PS.

Next, using the same range of *k*_ON-PS_ values, we simulated PS and PI(4)P transfer rates in a situation of lipid exchange between A and B membranes that initially contained 5 μM PS and PI(4)P, respectively (Fig. 5b, pink dots for PS and orange dots for PI(4)P). Acceleration factors were determined from rates established for each lipid in exchange and non-exchange contexts. The PS transfer rate found to be maximal at *k*_ON-PS_ = 3.7 μM^−1^ s^−1^ when PI(4)P was absent, slightly increased in the presence of PI(4)P (acceleration factor = 0.13). If *k*_ON-PS_ > 3.7 μM^−1^ s^−1^, the PS transfer rates were lower in a non-exchange situation but considerably higher if PI(4)P was present as counterligand. In contrast, for k_ON-PS_ < 3.7 μM^−1^ s^−1^, the PS transfer rate decreased toward zero, even if PI(4)P was present, and acceleration factors were almost null. In parallel, the PI(4)P transfer rates were found to be systematically higher in the presence of PS yet to a degree that depended on *k*_ON-PS_ values (Fig. 5b). Finally, we noted that an ORD efficiently exchanges PS and PI(4)P if it has a higher affinity for PS than PI(4)P (*k*_ON-PS_ > *k*_ON-PI(4)P_, Fig. 5b, gray area). These simulations again corroborated our experimental data. In non-exchange situations, saturated PS species, which are low-affinity ligands compared to 16:0/16:0-PI(4)P, are transferred at the fastest rates; yet these rates barely or marginally increase once PI(4)P is present. In contrast, unsaturated PS species, which are globally better ligands than 16:0/16:0-PI(4)P, are slowly transferred in non-exchange situations, but much faster when PI(4)P is present. In all cases, the PI(4)P transfer rate is unchanged or higher in the presence of PS.

To consolidate this analysis, we plotted the acceleration factors for PS and PI(4)P, simulated for different *k*_ON-PS_ and *k*_ON-PI(4)P_ values (Additional file 1: Fig. S6a), against each other and compared the curves obtained with experimental factors shown in Fig. 1e. By setting *k*_ON-PI(4)P_ at 40 or 75 μM^−1^ s^−1^, we obtained curves that follow the distribution of acceleration factors obtained with ORD8 and Osh6p, respectively (Fig. 5c).

Finally, simulations performed with variable *k*_ON-PI(4)P_ values (10, 40, 75, or 100 μM^−1^ s^−1^) indicated that, when the ORD had a higher affinity for PI(4)P, the PI(4)P transfer rate decreased in a non-exchange context (dashed lines, Additional file 1: Fig. S6a) but increased to a greater extent in a situation of PS/PI(4)P exchange. Interestingly, a different picture emerged if the affinity of the ORD for PI(4)P was increased by ten, by lowering the *k*_OFF-PI(4)P_ value from 1 to 0.1 s^−1^ instead of increasing the *k*_ON-PI4P_ value from 10 to 100 μM^−1^ s^−1^ (Additional file 1: Fig. S6b): PI(4)P transfer rates were low in non-exchange conditions and only slightly higher in exchange conditions, for all tested *k*_ON-PS_ values. PS transfer was more rapid in exchange conditions with high *k*_ON-PS_ values. This resembled our data showing that unsaturated PI(4)P species were poorly transferred and exchanged for PS while PS transfer was enhanced by these PI(4)P species (Fig. 2). Overall, our model showed how variations in the capacity of the ORD to extract and deliver PS and PI(4)P could modify how it transfers and exchanges these lipids.

### Osh6p and ORD8 cannot use PI(4,5)P_2_ if PI(4)P is present in membranes

**Fig. 6:**
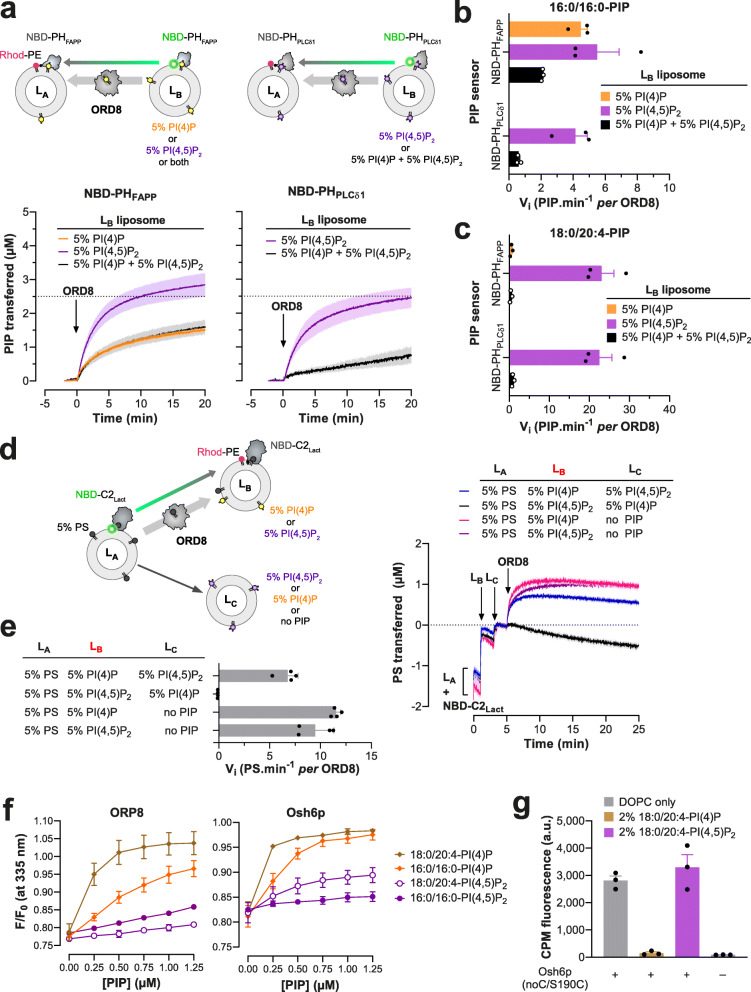
PIP selectivity of Osh6p and ORD8. **a** Intermembrane PI(4)P and PI(4,5)P_2_ transfer activity of ORD8 measured with donor *L*_B_ liposomes containing either PI(4)P or PI(4,5)P_2_ or both PIPs. *L*_B_ liposomes (200 μM total lipid concentration) made of DOPC and containing either 5% 16:0/16:0-PI(4)P or 5% 16:0/16:0-PI(4,5)P_2_ or both PIPs were mixed with NBD-PH_FAPP_ (250 nM) in HK buffer at 37 °C. After 1 min, *L*_A_ liposomes, made of DOPC and containing 2% Rhod-PE (200 μM), were added to the reaction mix. After 3 min, ORD8 was injected (240 nM). Similar experiments were conducted with NBD-PH_PLCδ1_ (250 nM) and *L*_B_ liposomes containing only 5% 16:0/16:0-PI(4,5)P_2_ or also 5% 16:0/16:0-PI(4)P. PIP transport was followed by measurement of the quenching of the fluorescence signal of NBD-PH_PLCδ1_ or NBD-PH_FAPP_ caused by the translocation of the lipid sensor from *L*_B_ to *L*_A_ liposomes and FRET with Rhod-PE. The signal was normalized in term of PI(4)P or PI(4,5)P_2_ amount delivered into *L*_A_ liposomes. The injection of ORD8 set time to zero. Each trace is the mean ± s.e.m. of independent kinetics (*n* = 3). **b** Initial 16:0/16:0-PIP transfer rates determined with NBD-PH_FAPP_ and NBD-PH_PLCδ1_. Data are represented as mean ± s.e.m. (error bars, *n* = 3). **c** Initial rates of 18:0/20:4-PIP transfer between membranes determined for ORD8 using NBD-PH_FAPP_ and NBD-PH_PLCδ1_ sensors. Data are represented as mean ± s.e.m. (error bars, *n* = 3). **d** *L*_A_ liposomes (200 μM total lipid), made of DOPC and 16:0/18:1-PS (95:5), were mixed with NBD-C2_Lact_ in HK buffer at 37 °C. After 1 min, *L*_B_ liposomes (200 μM), consisting of 93% DOPC, 2% Rhod-PE, and 5% 18:0/20:4-PI(4)P or 18:0/20:4-PI(4,5)P_2_, were injected. Two minutes later, a third population of liposomes (*L*_C,_ 200 μM) made of DOPC, doped or not with 5% 18:0/20:4-PI(4)P or 18:0/20:4-PI(4,5)P_2_, was injected. ORD8 (240 nM final concentration) was injected 2 min later. PS delivery into *L*_B_ liposomes was followed by measurement of the quenching of the fluorescence of NBD-C2_Lact_ provoked by its translocation from *L*_A_ to *L*_B_ liposomes and FRET with Rhod-PE. The signal was normalized in term of PS amount transferred into *L*_B_ liposomes. **e** Initial rates of ORD8-mediated PS transfer into acceptor *L*_B_ liposomes, as a function of the presence of PI(4)P and PI(4,5)P_2_ in acceptor *L*_B_ and/or *L*_C_ liposomes. Data are represented as mean ± s.e.m. (error bars, *n* = 4). **f** Competition assay. DOPC/NBD-PS liposomes (98:2, 100 μM total lipid) were added to ORD8 or Osh6p (200 nM) in HK buffer at 30 °C. The sample was excited at 280 nm and the emission was measured at 335 nm. Incremental amounts of liposome, containing 5% PI(4)P or PI(4,5)P_2_, were injected to the reaction mix. The signal was normalized considering the initial *F*_max_ fluorescence, prior to the addition of NBD-PS-containing liposomes, and the dilution effect due to liposome addition. Data are represented as mean ± s.e.m. (*n* = 3). **g** Accessibility assay. CPM (4 μM) was mixed with 400 nM Osh6p(noC/S190C) in the presence of pure DOPC liposomes or liposomes doped with 2% 18:0/20:4-PI(4)P or 18:0/20:4-PI(4,5)P_2_. Intensity bars correspond to the fluorescence measured 30 min after adding CPM (*n* = 3)

ORP5/8 have been suggested, notably based on in vitro data, to use PI(4,5)P_2_ instead of PI(4)P as a counterligand to supply the PM with PS [17] but this conclusion is disputed [19]. To address this issue, we measured how ORD8 transferred PI(4)P and PI(4,5)P_2_ (with 16:0/16:0 acyl chains) from *L*_B_ liposomes that contained only one kind of PIP or, like the PM, both PIPs, to *L*_A_ liposomes. NBD-PH_FAPP_, which can detect PI(4,5)P_2_ in addition to PI(4)P [17], was used as sensor. ORD8 transported PI(4,5)P_2_ more swiftly than PI(4)P (Fig. 6a, b), as previously shown, but surprisingly, when both PIPs were in *L*_B_ liposomes, the transfer kinetics was comparable to that measured with PI(4)P alone.

To understand why, we specifically measured the transfer of PI(4,5)P_2_ with a sensor based on the PH domain of the phospholipase C-δ1 (PH_PLCδ1_), which has a high affinity and specificity for the PI(4,5)P_2_ headgroup [43]. This domain was reengineered to include, near its PI(4,5)P_2_-binding site [44], a unique solvent-exposed cysteine (C61) to which a NBD group was attached (Additional file 1: Fig. S7a). In flotation assays this NBD-PH_PLCδ1_ construct associated with liposomes doped with PI(4,5)P_2_ but not with liposomes only made of DOPC or containing either PI or PI(4)P (Additional file 1: Fig. S7b). Fluorescence assays also indicated that NBD-PH_PLCδ1_ bound to PI(4,5)P_2_-containing liposomes, as its NBD signal underwent a blue-shift and a 2.2-fold increase in intensity (Additional file 1: Fig. S7c). A binding curve was established by measuring this change as a function of the incremental addition of these liposomes (Additional file 1: Fig. S7d, Additional file 2: Table S7). In contrast, no signal change occurred with PI(4)P-containing liposomes, indicative of an absence of binding (Additional file 1: Fig. S7c, d, Additional file 2: Table S7). NBD-PH_PLCδ1_ was thus suitable to detect PI(4,5)P_2_ but not PI(4)P. It was substituted for NBD-PH_FAPP_ to measure to what extent ORD8 specifically transported PI(4,5)P_2_ from *L*_B_ liposomes, containing or not PI(4)P, to *L*_A_ liposomes. Remarkably, we found that ORD8 efficiently transferred PI(4,5)P_2_ but only if PI(4)P was absent (Fig. 6a,b). Similar conclusions were reached using each PIP sensor by assaying ORD8 with PI(4)P and PI(4,5)P_2_ ligands with 18:0/20:4 acyl chains (Fig. 6c, Additional file 1: Fig. S8a), and Osh6p using PIPs with a 16:0/16:0 composition (Additional file 1: Fig. S8b, c). These data indicate that ORP8 and Osh6p preferentially extract PI(4)P from a membrane that contains both PI(4)P and PI(4,5)P_2_, suggesting that they use PI(4)P rather than PI(4,5)P_2_ in exchange cycles with PS at the PM.

To address this possibility in vitro, we devised an assay with three liposome populations (Fig. 6d, e) to examine whether ORP8 delivers PS in a PI(4)P-containing membrane or in a PI(4,5)P_2_-containing membrane. First, *L*_A_ liposomes doped with 5% PS were mixed with NBD-C2_Lact_. Then *L*_B_ liposomes, containing 5% PI(4)P and 2% Rhod-PE, and *L*_C_ liposomes, only made of PC, were successively added. Injecting ORD8 provoked a quenching of the NBD signal, indicating that the C2_Lact_ domain moved onto the *L*_B_ liposomes. The signal normalization indicated that ~ 1 μM of PS was transferred to *L*_B_ liposomes. Equivalent data were obtained with *L*_C_ liposomes doped with PI(4,5)P_2_, suggesting that this lipid has no influence on the PI(4)P-driven transfer of PS to *L*_B_ liposomes mediated by ORD8. We performed mirror experiments with *L*_B_ liposomes that contained PI(4,5)P_2_ and *L*_C_ liposomes with or without PI(4)P. Remarkably, PS was transferred to *L*_B_ liposomes but not if *L*_C_ liposomes contained PI(4)P. We concluded that ORP8 selectively delivers PS in a compartment that harbors PI(4)P if PI(4,5)P_2_ is present in a second compartment. This suggests that PI(4)P, and not PI(4,5)P_2_, is used by ORP5/8 to transfer PS intracellularly.

These observations suggest that ORD8 and Osh6p have a lower affinity for PI(4,5)P_2_ than for PI(4)P. Confirming this, the NBD-PS-based competition assay showed that each protein barely bound to PI(4,5)P_2_ compared to PI(4)P (with 16:0/16:0 or 18:0/20:4 acyl chains, Fig. 6f and Additional file 2: Table S4). Likewise, TSAs indicated that Osh6p incubated with liposomes containing 16:0/16:0- and 18:0/20:4-PI(4)P or PI(4,5)P_2_ was loaded with and stabilized by PI(4)P but not PI(4,5)P_2_ (Additional file 1: Fig. S8d, e and Additional file 2: Table S8). Finally, we evaluated the conformational state of Osh6p in the presence of each PIP. To this end, we used a version of the protein, Osh6p(noC/S190C), which has a unique cysteine at position 190; this residue is solvent-exposed only if the molecular lid that controls the entrance of the binding pocket of the protein is open [45]. This construct was added to liposomes devoid of PIPs or containing 2% PI(4)P or PI(4,5)P_2_. Then, 7-diethylamino-3-(4′-maleimidylphenyl)-4-methylcoumarin (CPM), a molecule that becomes fluorescent only when forming a covalent bond with accessible thiol, was added to each sample. After a 30-min incubation, a high fluorescence signal was measured with Osh6p(noC/S190C) mixed with PC liposomes, indicating that the protein remained essentially open over time (Fig. 6g). In contrast, almost no fluorescence was recorded when the protein was incubated with PI(4)P-containing liposomes, indicating that it remained mostly closed, as previously shown [45]. Remarkably, a high signal was obtained with liposomes doped with 2% PI(4,5)P_2_, indicating that Osh6p remained open as observed with pure PC liposomes. Altogether, these data suggest that Osh6p/7p and ORP5/8 have a low affinity for PI(4,5)P_2_ compared to PI(4)P, likely as they cannot form stable and closed complexes with this lipid.

### Sterol abundance in membrane does not enhance PS delivery and retention

**Fig. 7:**
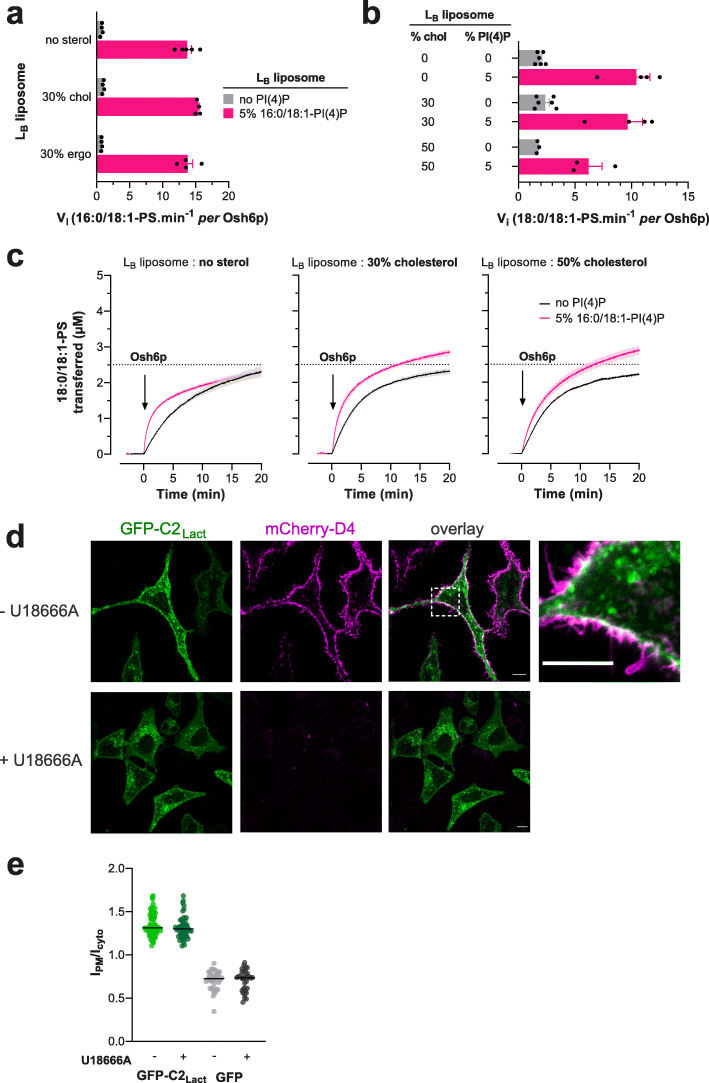
Influence of the sterol content of acceptor membranes on PS delivery. **a** Initial rates of 16:0/18:1-PS transfer measured with Osh6p (200 nM) at 30 °C from *L*_A_ (5% PS) to *L*_B_ liposomes with different bulk lipid compositions and containing or not 5% 16:0/18:1-PI(4)P (at the expense of PC). Data are represented as mean ± s.e.m. (non-exchange condition, *n* = 4–5, exchange condition, *n* = 4). **b** Initial rates of 18:0/18:1-PS transfer from *L*_A_ to *L*_B_ liposomes without or with 30% or 50% cholesterol, and containing or not 5% 16:0/18:1-PI(4)P. Data are represented as mean ± s.e.m. (*n* = 3–6). **c** Experiments are similar to those shown in **Fig. S9a ** except that 16:0/18:1-PS was replaced by 18:0/18:1-PS in *L*_A_ liposomes. *L*_B_ liposomes contained 0, 30, or 50% cholesterol. Each trace is the mean ± s.e.m. of kinetics recorded in independent experiments (*n* = 3–6). **d** Confocal images of live GFP-C2_Lact_-expressing HeLa cells (green) treated or not with U18666A for 24 h at 37 °C. Cholesterol in the PM was detected by incubating the cells for 10 min with mCherry-D4 (purple) at room temperature and then washed with medium prior to imaging. The overlay panel shows merged green and magenta images. The top-right image corresponds to a higher magnification (× 5.2) image of the area outlined in white. Scale bars: 10 μm. **e** Quantification of the ratio of GFP-C2_Lact_ signal at the PM to the cytosolic GFP-C2_Lact_ signal, as assessed by wide-field microscopy and line scan analysis. GFP-C2_Lact_-expressing HeLa cells with or without U18666A treatment (2.5 μg/mL for 24 h at 37 °C) as shown in **d** (mean ± s.e.m., *n* = 68 cells for non-treated cells and *n* = 51 cells for treated cells; data are pooled from four independent experiments for each condition). Control experiments were done with GFP-expressing HeLa cells (mean ± s.e.m., *n* = 37 cells for non-treated cells and *n* = 33 cells for treated cells; data are pooled from two independent experiments for each condition)

Like PS, sterol is synthesized in the ER and enriched in the PM, where it constitutes 30–40% of all lipids [46]. PS is thought to associate laterally with sterol, thus retaining sterol in the inner leaflet of the PM and controlling its transbilayer distribution [30, 31]. However, it was not known whether sterol stabilizes PS and thereby aids ORP/Osh proteins to accumulate PS in the PM. To explore this possibility, we measured in vitro the speed at which Osh6p transported PS from *L*_A_ liposomes to *L*_B_ liposomes, containing or not 30% cholesterol or ergosterol, and doped or not with 5% PI(4)P. These assays were performed using 16:0/18:1-PS and 16:0/18:1-PI(4)P, as in yeast PM, these predominant PS and PI(4)P species are thought to co-distribute in the presence of sterol [22]. However, we observed that the transfer of PS was not markedly impacted by higher contents of sterol in *L*_B_ liposomes in non-exchange and exchange conditions (Fig. 7a, Additional file 1: Fig. S9a). We then replaced 16:0/18:1-PS in our assays with 18:0/18:1-PS shown to segregate with cholesterol in vitro [31]. Yet, with *L*_B_ liposomes containing 0, 30 or even 50% cholesterol, no change was seen in the 18:0/18:1-PS transfer rate in a non-exchange context. If PI(4)P was present in *L*_B_ liposomes, PS was transferred more rapidly but at a slightly lesser extent when *L*_B_ liposomes also contained 50% cholesterol (Fig. 7b,c). This suggests that sterols do not favor PS transfer, possibly as they are dispensable for PS retention. To examine more extensively how cholesterol controls the retention of PS in the complex context of the PM, we used a GFP-C2_Lact_ probe to examine whether the steady-state accumulation of PS in the PM of HeLa cells was impacted when cholesterol was depleted from the PM. This depletion was achieved by treating the cells for 24 h with U18666A, a compound that blocks lysosomal-to-PM sterol movement through the inhibition of Niemann-Pick disease, type C1 (NPC1) protein [31, 47, 48]. Such a treatment lowers sterol levels without provoking the remodeling of the PM that occurs following a faster and acute sterol removal [30]. Using the sterol sensor mCherry-D4, we detected sterol in the PM of untreated cells but not in U18666A-treated cells (Fig. 7d). The same results were obtained with a mCherry-D4H construct (carrying the D434S mutation) even if it can detect lower sterol density [31], confirming that the PM was highly deprived in sterol (Additional file 1: Fig. S9b). However, no change was seen in the distribution of PS, which remained in the PM. This was ascertained by measuring the relative distribution of GFP-C2_Lact_ between the PM and the cytosol, in treated and untreated cells, using Lyn11-FRB-mCherry as a stable PM marker and internal reference (Fig. 7e, Additional file 1: Fig. S9c). We conclude that a high, normal level of sterol in the PM is not critical for PS retention and for PS/PI(4)P exchange. However, because we primarily impacted a NPC1-regulated pool of sterol, and as D4 and D4H probes do not detect sterol below a certain threshold [49], we cannot rule out that a sterol pool, inaccessible to our depletion procedure, contributes to stabilizing PS in the PM.

## Discussion

LTPs have been discovered and studied for more than 40 years, yet few studies have explored how their activity depends on the nature of the lipid acyl chains. A few kinetic studies have shown that a nonspecific-LTP transfers shorter PC more easily than long PC [50], that a glycolipid transfer protein (GLTP) preferentially transports short glucosylceramides [51] and that the ceramide transfer protein (CERT) is active with ceramide species whose length does not exceed the size of its binding pocket [52, 53]. Moreover, the link between the activity of LTPs and their affinity for lipid ligands remained largely obscure. Here, we measured how fast LTPs transfer saturated *vs* unsaturated lipids between membranes, both in a situation of simple transfer and in a situation of exchange with a second ligand, and we measured their relative affinity for these lipids. Our investigations used approaches that detect the transfer and binding capacities of LTPs with unmodified lipid ligands (i.e., without bulky fluorophores), thus with an unprecedented level of accuracy. This study offers novel insights into PS/PI(4)P exchangers and, by identifying how the activity of LTPs relates to their affinity for lipid ligands, provides general rules that serve to better analyze lipid transfer processes.

Our kinetic measurement indicated that overall ORD8 transfers PS and PI(4)P more slowly than Osh6p does. This might be due to structural differences between the two proteins or to the fact that the ORD of ORP8 only functions optimally in the context of the full-length protein or between closely apposed membranes. Apart from this, Osh6p and ORD8 responded similarly to the same changes in the PS and PI(4)P acyl chain structures. In particular, they transferred these lipids quite differently depending on whether they were saturated or not. This seems to be mainly because these LTPs have a lower affinity for saturated than unsaturated lipids. Remarkably, the presence of only one double bond in one of the acyl chains of the ligand is sufficient to significantly change their behavior. Our kinetic model suggests that the affinity of PS/PI(4)P exchangers for lipid ligands and their capacity to transfer them is governed by the extraction step (reflected by the *k*_ON_ values). An early study of large series of PE and PC species showed that increasing the degree of unsaturation in the acyl chains of phospholipids increases the rate at which they spontaneously desorb from the membrane [54]. One might therefore posit that the intrinsic propensity of PS species to move out of the membrane determines how they are captured and transferred by ORP/Osh proteins. However, several data suggest that the intrinsic tendency of PS species to leave the membrane cannot explain, or only very partially, why these lipids are more or less easily captured by Osh6p and ORD8.

First, considering the spontaneous desorption rates measured with other phospholipids [54], we should have obtained the exact same results with 18:0/18:1-PS as with 18:1/18:0-PS, which was not the case, notably in binding assays. Secondly, 16:0/16:0-PS should have been as good a ligand as an unsaturated species such as 16:0/18:1-PS and 18:1/18:1-PS whereas shorter saturated PS species (12:0/12:0 and 14:0/14:0) should have been better. Also, similar results should have been obtained with 16:0/16:0-PI(4)P and 16:0/18:1-PI(4)P. Structural analyses revealed that the sn-1 acyl chain of PS or PI(4)P is deeply inserted in the binding pocket, and that their sn-2 acyl chain is twisted, pointing its end to the lid that closes the pocket [12, 13]. These structural constraints along with other parameters, such as the intrinsic dynamics of lipid species in a bilayer, might govern how they are captured and stabilized by ORP/Osh proteins. In the future, studies addressing these hypotheses at the atomic level will be of great interest to understand what imparts these LTPs with such an enigmatic selectivity for unsaturated lipids. For instance, solving the structure of Osh6p or ORD8 in complex with various lipid species along with molecular dynamics (MD) simulations [55–57] might shed light on how lipid species are stabilized inside the binding pocket, released into or extracted from the membrane.

Kinetic data obtained with unsaturated lipids that correspond to or resemble PS and PI(4)P species that are prominent in yeast and human cells, provided hints on the activity of these LTPs in cellular context, which remains difficult to obtain in situ. Osh6p slowly transfers 16:0/18:1-PS and 16:0/18:1-PI(4)P, the most abundant yeast PS and PI(4)P species [22, 24, 28, 37], under non-exchange conditions and ten times faster under exchange condition, and with the same initial velocity (~ 14 lipid min^−1^). This suggests that, at the ER/PM interface, the transfer of PS by Osh6p/7p in yeast is highly dependent on the synthesis of PI(4)P in the PM and is tightly coupled with the transfer of PI(4)P in the opposite direction. Another PS species, 18:0/18:1-PS paired with 16:0/16:0-PI(4)P or 16:0/18:1-PI(4)P efficiently completes fast exchange, as does 18:1/18:1-PS with 16:0/16:0-PI(4)P. These PS species do not exist in yeast but resemble 16:0/18:1-PS and the few other major yeast PS species (16:0/16:1, 16:1/18:1, 16:1/16:1), which contain at least one monounsaturated acyl chain. In comparison, PS species with one or two di-unsaturated chains seemed less suitable for exchange processes. An equivalent analysis with PI(4)P remains difficult to conduct as much fewer PI(4)P species were assayed. The fact that polyunsaturated PI(4)P, the major brain PI(4)P species, exchanges poorly with 16:0/18:1-PS does not mean that Osh6p is specifically designed for yeast monounsaturated PI(4)P. Indeed, slow transfers were measured when assaying polyunsaturated PI(4)P with ORD8. Collectively, our data suggest that Osh6p can efficiently exchange unsaturated PS and PI(4)P species at the ER/PM interface. This supports the notion that Osh6p/7p regulate a pool of unsaturated PS and PI(4)P that together with ergosterol form functional lipid nanodomains [22]. Moreover, this is compatible with the observation that 34:1-PI(4)P (i.e., 16:0/18:1-PI(4)P) can be predominantly hydrolyzed by Sac1p [23]. However, our investigations suggest that the delivery of 16:0/18:1-PS in the PM, and its exchange for PI(4)P are likely not promoted by the presence of ergosterol. Finally, our data suggest that the massive increase in the proportion of 16:0/18:1-PS observed between the ER membrane and the PM [28] (from 30 to 60% compared to the other PS species) is not caused by a preferential transfer of this species by Osh6p/7p.

Analyzing the association of ORD8 with 16:0/18:1-PS and 18:0/18:1-PS, the prevalent PS species in human cells, and the speed at which it transfers these lipids, we can draw similar conclusions as those obtained with Osh6p. These PS species are recognized with high affinity by ORD8 and, interestingly, a substantial fraction of ORD8 can be purified as a stable complex with each of these two PS species from human cells [14]. Indeed, ORD8, purified from HEK293 cells, is bound to 34:1 and 36:1-PS that correspond to 16:0/18:1-PS and 18:0/18:1-PS, respectively, considering lipidomic analyses of this cell line [38]. The identification of these PS species as ligands is likely due to their cellular abundance but also their high affinity for ORD8. In vitro, these PS species are slowly transferred under non-exchange conditions and faster under exchange conditions to an extent that is however only slightly dependent on the PI(4)P species used as counterligand (16:0/16:0, 18:1/18:1 or 18:0/20:4-PI(4)P). This suggests that the capacity of ORP5/8 to efficiently transfer cellular PS species strongly depends on PI(4)P but is minimally influenced by the unsaturation degree of this latter. In line with this, when ORD8 is purified from cells, it is also found partially loaded with 36:1 and 36:2-PI(4)P [14] (likely 18:0/18:1-PI(4)P and 18:0/18.2-PI(4)P [38]), which might indicate that this domain can use unsaturated PI(4)P species other than those tested in our assays for PS/PI(4)P exchange. In parallel, we noted that, when exchange is possible, the transfer of 18:0/20:4-PI(4)P and 18:1/18:1-PI(4)P is faster but remains much slower than the PS transfer, suggesting a weak coupling between the two transfer processes. This presumably arises from the high affinity of the ORD for these PI(4)P species, as suggested by our kinetic model. It has been reported that the hydrolysis of PI(4)P by Sac1 is mandatory for OSBP-mediated sterol/PI(4)P exchange [58]. Indeed, OSBP has a much higher affinity for PI(4)P than for sterol and the hydrolysis of PI(4)P by Sac1, by mass action, promotes the exchange process. Possibly, Sac1 also enhances the transfer of PI(4)P from the PM to the ER by ORP5/8 while facilitating the extraction of PS from this organelle, and thus improves the coupling between PS and PI(4)P transfer at the ER/PM interface. This model would be valid whatever the nature of PI(4)P subspecies as Sac1 can hydrolyze mono-, di-, and polyunsaturated PI(4)P with a similar efficiency [41]. Collectively, these observations suggest that the degree of unsaturation of PI(4)P, a parameter that varies between cells in tissues and cultured transformed cells [32], should not strongly impact the PS/PI(4)P exchange capacity of ORP5/8. One can thus envision that ORP5/8-mediated exchange cycles are not linked with the preferential synthesis and consumption of certain PIP subspecies contrary to the PI cycle [33]. Finally, we measured in vitro that cholesterol does not promote the delivery of 18:0/18:1-PS in a membrane and that depleting sterol from the PM of human cells does not perturb the cellular distribution of PS. This suggests that a normal and high level of sterol in the PM does not seem critical for the accumulation of PS in that membrane. However, considering the existence of different sterol pools in the PM [59] and that the D4 and D4H probes do not detect sterol below a certain threshold concentration [49], we cannot exclude the possibility that a substantial amount of PS is retained by a small pool of sterol that cannot be removed by our depletion procedure. In the future, lipid transfer assays in primary and transformed cells coupled to lipidomic analyses will be of great value to more precisely define the dependency of ORP5/8 activity on the nature of PI(4)P and sterol.

It was unclear whether ORP5/8 could use PI(4,5)P_2_ instead of PI(4)P as a counterligand [14, 17, 19]. Corroborating previous observations [17], we measured that Osh6p and ORD8 transferred PI(4,5)P_2_ between liposomes more rapidly than PI(4)P. However, when PI(4)P and PI(4,5)P_2_ both resided in the same donor membrane, only PI(4)P was transferred to acceptor membranes, for all tested acyl chain compositions. Moreover, only PI(4)P was used as counterligand for the transfer of PS when both PIPs were present. In fact, Osh6p and ORD8 show a much lower affinity for PI(4,5)P_2_ than for PI(4)P. This likely relates to the fact that PI(4,5)P_2_, contrary to PI(4)P (or PS), cannot be entirely buried in the pocket and capped by the lid. This hypothesis was suggested by structural analyses of ORP1 and ORP2 in complex with PI(4,5)P_2_ [35, 36] and confirmed by our in vitro assays. Because PI(4)P and PI(4,5)P_2_ co-exist in the PM, this strongly suggests that only PI(4)P is used by ORP5 or ORP8 for the exchange with PS in the cell. Our data also suggest that ORP1, ORP2, and ORP4L [60] cannot trap PIPs other than PI(4)P in cells or only if they operate on organelle membranes devoid of PI(4)P.

Quite interestingly, the comparison between the transfer rates and affinity determined with Osh6p and ORD8 for various lipid species allows us to infer general rules that can serve to better understand the cellular activity of LTPs. A first lesson is that, in a simple transfer process between membranes, a low-affinity ligand can be transferred more rapidly down its concentration gradient than a high-affinity ligand. This was observed when comparing saturated PS with unsaturated PS or PI(4,5)P_2_ with PI(4)P. Presumably, as suggested by our kinetic model, it lies in the fact that a low-affinity recognition process can be detrimental when the ligand is extracted from a donor membrane but an advantage in the transfer process, notably by preventing any re-extraction of the ligand from the acceptor membrane.

The picture is different with a membrane system of higher complexity that reconstitutes a cellular context more faithfully. In exchange conditions, the ability of Osh6p and ORD8 to transport saturated PS was poorly enhanced or often inhibited when PI(4)P was present as a counterligand, because these LTPs have a higher affinity for the latter. When PI(4)P and PI(4,5)P_2_ were present in the same donor membrane, these LTPs preferentially transferred PI(4)P, for which they have the highest affinity, to acceptor membranes. In an even more complex system where PS, PI(4)P, and PI(4,5)P_2_ were present each in distinct liposome populations, mimicking three cellular compartments, ORD8 used PI(4)P as a counterligand to transfer PS between two membranes.

These observations have important implications. They suggest first that some caution must be exercised when analyzing in vitro data using membranes of low compositional complexity: measuring a fashe same results were obtainedt transfer rate for a given lipid ligand and an LTP does not necessarily mean that this ligand is the true cellular cargo of the LTP. When considering a cellular context, one can assume that a mere lipid transporter preferentially recognizes and transfers its high-affinity lipid ligand. This potentially implies, as suggested in vitro, a lower speed of transfer but at the benefit of a higher accuracy as no fortuitous ligand can be taken. However, this limitation in terms of speed is lifted if the LTP can exchange this high-affinity ligand for a second one, as measured with Osh6p and ORP8 using unsaturated PS and PI(4)P. This can primarily be explained by the fact that this second ligand prevents the re-extraction by the LTPs of the other ligand from its destination compartment, which improves its net delivery. These observations on Osh6p and ORP8 confirm very first data on the sterol/PI(4)P exchange capacity of Osh4p [34]. Of note, our experiments and models suggest that an optimal exchange occurs, i.e., with similar PS and PI(4)P transfer rates along opposite directions, when a lipid exchanger has a similar affinity for each ligand. Interestingly, the experiments with PS, PI(4)P and PI(4,5)P_2_ even suggest that this exchange process can channel a lipid flux between two membrane-bound compartments if there are more than two compartments, such as in a cell. Collectively, our study supports the notion that lipid exchange processes are mechanisms that ensure fast, accurate, and directional transfer of lipids between organelles.

## Conclusion

Our study provides novel insights into PS/PI(4)P exchangers, by showing that their activity is highly dependent on whether their lipid ligands are saturated or not and indicating the extent to which they can regulate the acyl chain composition of the PM. Moreover, it establishes that these LTPs cannot control PM PI(4,5)P_2_ level by a direct PS/PI(4,5)P_2_ exchange mechanism and that, unexpectedly, their activity is not influenced by the sterol abundance in the PM. Importantly, by conducting both kinetics and binding measurements with a large set of lipids, we also determine how the activity of LTPs depends on their affinity for ligands and propose general mechanistic rules on intracellular lipid transport.

## Methods

### Protein expression, labelling, and purification

Osh6p, Osh6p(noC/S190C), NBD-PH_FAPP_, and NBD-C2_Lact_ were purified as previously described [13, 45, 61].Their concentration was determined by UV spectrometry.

GST-ORD8 (GST-ORP8[370-809]) [14] was expressed in *E. coli* (BL21-GOLD(DE3)) competent cells (Stratagene) grown in Luria Bertani Broth (LB) medium at 18 °C overnight upon induction with 0.1 mM isopropyl β-D-1-thiogalactopyranoside (IPTG). When the optical density of the bacterial suspension, measured at 600 nm (OD_600_), reached a value of 0.6, bacteria cells were harvested and re-suspended in cold buffer (50 mM Tris, pH 8, 500 mM NaCl, 2 mM DTT) supplemented with 1 mM PMSF, 10 μM bestatin, 1 μM pepstatin A, and cOmplete EDTA-free protease inhibitor tablets (Roche). Cells were lysed in a Cell Disruptor TS SERIES (Constant Systems Ltd.) and the lysate was centrifuged at 186,000×*g* for 1 h. Then, the supernatant was applied to Glutathione Sepharose 4B (Cytiva) for 4 h at 4 °C. After three washing steps with the buffer devoid of protease inhibitors, the beads were incubated with PreScission Protease (Cytiva) overnight at 4 °C to cleave off the ORD8 from the GST domain. The protein was recovered in the supernatant after several cycles of centrifugation and washing of the beads, concentrated, and injected onto a XK-16/70 column packed with Sephacryl S-200 HR to be purified by size-exclusion chromatography. The fractions with ~ 100% pure ORD8 were pooled, concentrated, and supplemented with 10% (v/v) pure glycerol (Sigma). Aliquots were prepared, flash-frozen in liquid nitrogen and stored at − 80 °C. The concentration of the protein was determined by measuring its absorbance at *λ* = 280 nm (*ε* = 81,820 M^−1^ cm^−1^).

To prepare NBD-labelled PH_PLCδ1_, an endogenous, solvent-exposed cysteine at position 48 of a GST-PH_PLCδ1_ construct [62] (PH domain of 1-phosphatidylinositol 4,5-bisphosphate phosphodiesterase delta-1, *R. norvegicus*, Uniprot: P10688) was replaced by a serine, and a serine at position 61 was replaced by a cysteine using the Quikchange kit (Agilent). GST-PH_PLCδ1_ was expressed in *E. coli* BL21-GOLD(DE3) competent cells at 20 °C for 24 h upon induction with 0.1 mM IPTG (at OD_600_ = 0.6). Harvested bacteria cells were re-suspended in 50 mM Tris, pH 7.4, 120 mM NaCl buffer containing 2 mM DTT and supplemented with 1 mM PMSF, 10 μM bestatin, 1 μM pepstatin A, and EDTA-free protease inhibitor tablets. Cells were lysed and the lysate was centrifuged at 186,000×*g* for 1 h. Next, the supernatant was applied to Glutathione Sepharose 4B for 4 h at 4 °C. The beads were washed three times with protease inhibitor-free buffer and incubated with thrombin at 4 °C for 16 h to cleave off the protein from the GST domain. Then, the eluate obtained after thrombin treatment was concentrated and mixed (after DTT removal by gel filtration on illustra NAP-10 columns (Cytiva) with a 10-fold excess of N,N′-dimethyl-N-(iodoacetyl)-N′-(7-nitrobenz-2-oxa-1,3-diazol-4-yl) ethylenediamine (IANBD-amide, Molecular Probes). After 90 min on ice, the reaction was stopped by adding a 10-fold excess of L-cysteine over the probe. The free probe was removed by size-exclusion chromatography using a XK-16/70 column packed with Sephacryl S-200 HR. The fractions that contained NBD-PH_PLCδ1_ were pooled, concentrated, and supplemented with 10% (v/v) pure glycerol. Aliquots were stored at − 80 °C once flash-frozen in liquid nitrogen. The labelled protein was analyzed by SDS-PAGE and UV-visible spectroscopy. The gel was directly visualized in a fluorescence imaging system (FUSION FX fluorescence imaging system) to detect NBD-labelled protein excited in near-UV and then stained with SYPRO Orange to determine the purity of NBD-labelled protein. The labelling yield (~ 100%) was estimated from the ratio between the absorbance of the protein at *λ* = 280 nm (*ε* = 17,990 M^−1^ cm^−1^ for PH_PLCδ1_) and NBD at *λ* = 495 nm (*ε* = 25,000 M^−1^ cm^−1^). The concentration of the protein was determined by a BCA assay and UV spectrometry.

mCherry-D4-His_6_ and mCherry-D4H-His_6_ (carrying the D434S mutation) were each overexpressed overnight in *E. coli* (BL21-GOLD(DE3)) at 18 °C for 20 h upon induction by 0.4 mM IPTG at OD_600_ = 0.6. Bacteria cells were harvested and re-suspended in 50 mM NaH_2_PO_4_/Na_2_HPO_4_, pH 8, 300 mM NaCl, 10 mM imidazole buffer supplemented with 1 mM PMSF, 10 μM bestatine, 1 μM pepstatine, and EDTA-free protease inhibitor tablets. Cells were broken and the lysate was centrifuged at 186,000×*g* for 1 h. Then, the supernatant was applied to HisPur Cobalt Resin (Thermo Scientific) for 4 h at 4 °C. The beads were loaded into a column and washed four times with buffer devoid of protease inhibitors. Bound protein was eluted from the beads incubated for 10 min with 20 mM NaH_2_PO_4_/Na_2_HPO_4_, pH 7.4, 250 mM imidazole buffer. This step was repeated six times to collect a maximal amount of protein. Each protein was concentrated and stored at − 80 °C in the presence of 10% (v/v) glycerol. The concentration of protein was determined by measuring the absorbance at *λ* = 280 nm (ε = 77,810 M^−1^ cm^−1^).

### Lipids

18:1/18:1-PC (1,2-dioleoyl-*sn*-glycero-3-phosphocholine or DOPC), 12:0/12:0-PS (1,2-dilauroyl-*sn*-glycero-3-phospho-L-serine or DLPS), 14:0/14:0-PS (1,2-dimyristoyl-*sn*-glycero-3-phospho-L-serine or DMPS), 16:0/16:0-PS (1,2-dipalmitoyl-*sn*-glycero-3-phospho-L-serine or DPPS), 18:0/18:0-PS (1,2-distearoyl-*sn*-glycero-3-phospho-L-serine or DSPS), 16:0/18:1-PS (1-palmitoyl-2-oleoyl-*sn*-glycero-3 phospho-L-serine or POPS), 18:0/18:1-PS (1-stearoyl-2-oleoyl-*sn*-glycero-3-phospho-L-serine or SOPS), 18:1/18:0-PS (1-oleoyl-2-stearoyl-*sn*-glycero-3-phospho-L-serine or OSPS), 18:1/18:1-PS (1,2-dioleoyl-*sn*-glycero-3-phospho-L-serine or DOPS), 16:0/18:2-PS (1-palmitoyl-2-linoleoyl-*sn*-glycero-3-phospho-L-serine), 18:2/18:2-PS (1,2-dilinoleoyl-*sn*-glycero-3-phospho-L-serine), liver PI (L-α-phosphatidylinositol, bovine), 16:0/18:1-PI(4)P (1-palmitoyl-2-oleoyl-*sn*-glycero-3-phospho-(1'-myo-inositol-4'-phosphate)), 18:1/18:1-PI(4)P (1,2-dioleoyl-*sn*-glycero-3-phospho-(1'-myo-inositol-4'-phosphate)), brain PI(4)P (L-α-phosphatidylinositol 4-phosphate), brain PI(4,5)P_2_ (L-α-phosphatidylinositol 4,5-bisphosphate), Rhodamine-PE (1,2-dipalmitoyl-*sn*-glycero-3-phosphoethanolamine-N-(lissamine rhodamine B sulfonyl)), 16:0/12:0 NBD-PS (1-palmitoyl-2-(12-[(7-nitro-2-1,3-benzoxadiazol-4-yl)amino]dodecanoyl)-*sn*-glycero-3-phosphoserine), 16:0/12:0 NBD-PC (1-palmitoyl-2-(12-[(7-nitro-2-1,3-benzoxadiazol-4-yl)amino]dodecanoyl)-*sn*-glycero-3-phosphocholine), cholesterol, and ergosterol were purchased from Avanti Polar Lipids. Saturated PIPs, namely 16:0/16:0-PI(4)P (1,2-dipalmitoyl-*sn*-glycero-3-phospho-(1'-myo-inositol-4'-phosphate) and 16:0/16:0-PI(4,5)P_2_ (1,2-dipalmitoyl-*sn*-glycero-3-phospho-(1′-myo-inositol-4′,5′-bisphosphate)) were purchased from Echelon Biosciences.

### Liposome preparation

Lipids stored in stock solutions in CHCl_3_ or CHCl_3_/methanol were mixed at the desired molar ratio. The solvent was removed in a rotary evaporator under vacuum. If the flask contained a mixture with PI(4)P and/or PI(4,5)P_2_, it was pre-warmed at 33 °C for 5 min prior to creating a vacuum. The lipid film was hydrated in 50 mM HEPES, pH 7.2, 120 mM K-Acetate (HK) buffer to obtain a suspension of multi-lamellar vesicles. The multi-lamellar vesicles suspensions were frozen and thawed five times and then extruded through polycarbonate filters of 0.2 μm pore size using a mini-extruder (Avanti Polar Lipids). Liposomes were stored at 4 °C and in the dark when containing fluorescent lipids and used within 2 days.

### Lipid transfer assays with two liposome populations

Lipid transfer assays were carried out in a Shimadzu RF 5301-PC or a JASCO FP-8300 spectrofluorometer. Each sample (600 μL final volume) was placed in a cylindrical quartz cuvette, continuously stirred with a small magnetic bar and thermostated at 30 °C and 37 °C for experiments done with Osh6p and ORD8, respectively. At precise times, samples were injected from stock solutions with Hamilton syringes through a guide in the cover of the spectrofluorometer. The signal of fluorescent lipid sensors (NBD-C2_Lact_, NBD-PH_FAPP_ and NBD-PH_PLCδ1_) was followed by measuring the NBD signal at *λ* = 530 nm (bandwidth 10 nm) upon excitation at *λ* = 460 nm (bandwidth 1.5 nm) with a time resolution of 0.5 s. To measure PS transfer, a suspension (540 μL) of *L*_A_ liposomes (200 μM total lipid, final concentration), made of DOPC and containing 5% PS and 2% Rhod-PE, was mixed with 250 nM NBD-C2_Lact_ in HK buffer. After 1 min, 30 μL of a suspension of *L*_B_ liposomes (200 μM lipids, final concentration) containing or not 5% PI(4)P was added to the sample. Three minutes after, Osh6p (200 nM) or ORD8 (240 nM) was injected. The amount of PS ([PS], expressed in μM) transferred over time was determined from the raw NBD traces using the formula [PS] = 2.5 × *F*_Norm_ with *F*_Norm_ = (*F* − *F*_0_)/(*F*_Eq_ − *F*_0-Eq_). *F* corresponds to the data point recorded over time. *F*_0_ is equal to the average NBD signal measured between the injection of *L*_B_ liposomes and LTP. *F*_Eq_ is the signal measured with the sensor in the presence of *L*_A-Eq_ and *L*_B-Eq_ liposomes, and *F*_0-Eq_ is the signal of the suspension of *L*_A-Eq_ only, prior to the addition of *L*_B__-__Eq_. The lipid composition of *L*_A-Eq_ and *L*_B-Eq_ liposomes were similar to that of *L*_A_ and *L*_B_ liposomes used in the transfer assays, except that both contained 2.5% PS (and additionally 2.5% PI(4)P in the experiments conducted in the context of the lipid exchange) to normalize the signal. The amount of PS transferred from *L*_A_ to *L*_B_ liposomes corresponds to 2.5 × *F*_Norm_, as one considers that at equilibrium one half of accessible PS molecules, contained in the outer leaflet of the *L*_A_ liposomes (i.e., corresponding to 5% of 0.5 × 200 μM total lipids) have been delivered into *L*_B_ liposomes.

To measure PI(4)P transfer, a suspension (540 μL) of *L*_B_ liposomes (200 μM total lipid) containing 5% PI(4)P was mixed with 250 nM NBD-PH_FAPP_ in HK buffer. After 1 min, 30 μL of a suspension of *L*_A_ liposomes (200 μM lipids) containing 2% Rhod-PE and doped or not with 5% PS were injected. After 3 additional minutes, LTP was injected. The amount of PI(4)P transferred from *L*_B_ to *L*_A_ liposomes was determined considering that [PI(4)P] = 2.5 × *F*_Norm_ with *F*_Norm_ = (*F* − *F*_0_)/(*F*_Eq_ − *F*_0_). *F* corresponded to the data point recorded over time, *F*_0_ was the average signal measured before the addition of LTP, and *F*_Eq_ was the average signal measured in the presence of *L*_A-Eq_ and *L*_B-Eq_ liposomes that each contained 2.5% PI(4)P (and additionally 2.5% PS to normalize data obtained in exchange conditions). At equilibrium, it is considered that one half of accessible PI(4)P molecules, contained in the outer leaflet of *L*_B_ liposomes, (i.e., corresponding to 5% of 0.5 × 200 μM total lipids) have been transferred into *L*_A_ liposomes. The transfer of PI(4,5)P_2_ from *L*_B_ to *L*_A_ liposomes was determined using NBD-PH_FAPP_ or NBD-PH_PLCδ1_ at 250 nM as described for PI(4)P transfer measurements except that *F*_Eq_ was determined using *L*_A_ and *L*_B_ liposomes that each contained 2.5% PI(4,5)P_2_. For all the measurements, the initial transport rates (or initial velocities) were determined from normalized curves by fitting the first eight data points (4 s) measured upon Osh6p/ORD8 injection with a linear function, divided by the amount of LTP and expressed in terms of lipid min^−1^ per protein.

### PS transport assay with three liposome populations

At time zero, *L*_A_ liposomes (200 μM total lipid, final concentration) containing 95% DOPC and 5% 16:0/18:1-PS were mixed with 250 nM NBD-C2_Lact_ in 480 μL of HK buffer at 37 °C in a quartz cuvette. After 1 min, 60 μL of a suspension of *L*_B_ liposomes (200 μM total lipid) made of 93% DOPC, 2% Rhod-PE, and 5% 18:0/20:4-PI(4)P or 18:0/20:4-PI(4,5)P_2_ were injected. After 2 additional minutes, 60 μL of a suspension of L_C_ liposomes (200 μM total lipid) consisting only of DOPC or containing 5% 18:0/20:4-PI(4)P or 18:0/20:4-PI(4,5)P_2_. Finally, after 2 min, ORD8 (240 nM) was injected. PS transport was followed by measuring the NBD signal at *λ* = 525 nm (bandwidth 5 nm) upon excitation at *λ* = 460 nm (bandwidth 1 nm) under constant stirring. The quenching of the NBD signal was due to the translocation of the probe onto *L*_B_ liposome doped with Rhod-PE and reflected how much PS was transferred from *L*_A_ to *L*_B_ liposomes. The amount of transferred PS (in μM) was determined by normalizing the NBD signal considering that [PS] = 2.5 × *F*_Norm_ with *F*_Norm_ = (*F* − *F*_0_)/(*F*_Eq_ − *F*_0_). *F* corresponded to data points measured over time, *F*_0_ corresponded to the NBD-C2_Lact_ signal measured when PS was only in *L*_A_ liposomes and *F*_Eq_ corresponded to the signal of the probe when PS was fully equilibrated between *L*_A_ and *L*_B_ liposomes. *F*_0_ value was obtained by averaging the fluorescence measured after the injection of *L*_C_ liposomes and before the injection of ORD8. *F*_Eq_ was the fluorescence of NBD-C2_Lact_ (250 nM) measured once it was sequentially mixed with L_A-Eq_ liposomes consisting of 97.5% DOPC and 2.5% 16:0/18:1-PS and L_B-Eq_ liposomes consisting of 95.5% DOPC, 2.5% 16:0/18:1-PS and 2% Rhod-PE, and L_C_ liposomes only made of DOPC (the concentration of each liposome population was 200 μM). Its value corresponded to the average fluorescence measured 15 min after the addition of *L*_C_ liposomes and for a 5-min period. *F*_Eq_ was also measured using only *L*_A_ and *L*_B_ liposomes and was found to be identical. A maximum of 2.5 μM of PS can be transferred from *L*_A_ to *L*_B_ liposomes as one half of accessible PS molecules, contained in the outer leaflet of the *L*_A_ liposomes, (i.e., 5% of 0.5 × 200 μM total lipids), can be transferred to reach equilibrium.

### NBD-PS-based competition assays

In a cylindrical quartz cuvette, Osh6p or ORD8 was diluted at 240 nM in a final volume of 555 μL of freshly degassed and filtered HK buffer at 30 °C under constant stirring. Two minutes after, 30 μL of a suspension of DOPC liposomes containing 2% NBD-PS was added (100 μM total lipid, 1 μM accessible NBD-PS). Five minutes after, successive injections of 3 μL of a suspension of DOPC liposomes enriched with a given PS or PI(4)P species (at 5%) were done every 3 min. Tryptophan fluorescence was measured at *λ* = 340 nm (bandwidth 5 nm) upon excitation at *λ* = 280 nm (bandwidth 1.5 nm). The signal was normalized by dividing *F*, the signal measured over time, by *F*_0_, the signal measured prior to the addition of the NBD-PS-containing liposome population, and corrected for dilution effects due to the successive injections of the second population of liposome. The signal between each liposome injection was averaged over 2 min to build the binding curve as a function of concentration of accessible non-fluorescent PS or PI(4)P species (from 0 to 1.25 μM).

### Thermal shift assay

The relative melting temperatures (*T*_m_) of Osh6p in an empty form or loaded with a lipid ligand were determined by measuring the unfolding of the protein as a function of increasing temperature through the detection of the denatured form of the protein by fluorescence. To prepare Osh6p-PS and Osh6p-PI(4)P complexes, the protein at 5 μM was incubated with heavy liposomes made of DOPC (800 μM total lipid), containing a given PS or PI(4)P subspecies (5%) and encapsulating 50 mM HEPES, pH 7.4, 210 mM sucrose buffer, in a volume of 250 μL of HK buffer. An apo form of the protein was prepared by incubating the protein with DOPC liposomes devoid of lipid ligands. Each sample was mixed by agitation for 30 min at 30 °C and then was centrifuged at 400,000×g for 20 min at 20 °C to pellet the liposomes using a fixed-angle rotor (Beckmann TLA 120.1). A fraction of each supernatant (200 μL) containing Osh6p loaded with lipid was collected and the concentration of each complex was assessed by measuring sample absorbance.

A volume of 15 μL of each Osh6p sample was mixed with 5× SYPRO Orange in an individual well of a 96-well PCR plate. The plates were sealed with an optical sealing tape (Bio-Rad) and heated in an Mx3005P Q-PCR system (Stratagene) from 25 to 95 °C with a step interval of 1 °C. The excitation and emission wavelengths were set at *λ* = 545 nm and *λ* = 568 nm, respectively (Cy3 signal). Fluorescence changes in the wells were measured with a photomultiplier tube. The melting temperatures (*T*_m_) were obtained by fitting the fluorescence data from 3 to 5 independent experiments with a Boltzmann model using the GraphPad Prism software.

### Flotation experiment

NBD-PH_PLCδ1_ protein (1 μM) was incubated with liposomes (1.5 mM total lipid) only made of DOPC, or additionally doped with 2% liver PI, 18:0/20:4-PI(4)P or 18:0/20:4-PI(4,5)P_2_, in 150 μL of HK buffer at room temperature for 10 min under agitation. The suspension was adjusted to 28% (w/w) sucrose by mixing 100 μL of a 60% (w/w) sucrose solution in HK buffer and overlaid with 200 μL of HK buffer containing 24% (w/w) sucrose and 50 μL of sucrose-free HK buffer. The sample was centrifuged at 240,000×*g* in a swing rotor (TLS 55 Beckmann) for 1 h. The bottom (250 μL), middle (150 μL), and top (100 μL) fractions were collected. The bottom and top fractions were analyzed by SDS-PAGE by direct fluorescence and after staining with SYPRO Orange, using a FUSION FX fluorescence imaging system.

### Fluorescence-based membrane binding assay

Measurements were taken in a 96-well black plate (Microplate 96 Well PS F-Bottom Black Non-Binding, Greiner Bio-one) using a TECAN M1000 Pro. Incremental amounts of DOPC liposomes, containing either 2% 18:0/20:4-PI(4)P or 18:0/20:4-PI(4,5)P_2_, were mixed with NBD-PH_PLCδ1_ (250 nM) at 25 °C in individual wells (100 μL final volume). NBD spectra were recorded from 509 to 649 nm (bandwidth 5 nm) upon excitation at 460 nm (bandwidth 5 nm). The intensity at *λ* = 535 nm was plotted as a function of total lipid concentration (from 0 to 300 μM).

### CPM accessibility assay

The day of the experiment, 100 μL from a stock solution of Osh6p(noC/S190C) construct was applied onto an illustra NAP-5 column (Cytiva) and eluted with freshly degassed HK buffer, according to the manufacturer’s indications to remove DTT. The concentration of the eluted protein was determined by UV spectroscopy considering *ε* = 55,810 M^−1^ cm^−1^ at *λ* = 280 nm. A stock solution of CPM (7-diethylamino-3-(4-maleimidophenyl)-4-methylcoumarin, Sigma-Aldrich) at 4 mg/mL was freshly prepared as described in [63] by mixing 1 mg of CPM powder in 250 μL of DMSO. Thereafter, this solution was diluted in a final volume of 10 mL of HK buffer and incubated for 5 min at room temperature. The solution was protected from light and used immediately. In individual wells of a 96-well black plate (Greiner Bio-one), Osh6p(noC/S190C) at 400 nM was mixed either with DOPC liposomes (400 μM total lipid) or liposomes containing 2% 18:0/20:4-PI(4)P or 18:0/20:4-PI(4,5)P_2_ in 200 μL of HK buffer. A small volume of CPM stock solution was then added to obtain a final concentration of 4 μM. After a 30 min incubation at 30 °C, emission fluorescence spectra were measured from 400 to 550 nm (bandwidth 5 nm) upon excitation at *λ* = 387 nm (bandwidth 5 nm) using a fluorescence plate reader (TECAN M1000 Pro). The maximal intensity of the spectral peak was at 460 nm. Control spectra were recorded in the absence of protein for each condition.

### Kinetic modeling

To analyze the experimental data, we considered that an ORD-mediated PS/PI(4)P exchange cycle can be described by the following sequence of reactions:

$$ O+{M}_A\ {\displaystyle \begin{array}{c}\overset{k_{\mathrm{ON}- Mb}}{\to}\\ {}\underset{k_{\mathrm{OFF}- Mb}}{\leftarrow}\end{array}}\ O-{M}_A $$

$$ O+{M}_B\ {\displaystyle \begin{array}{c}\overset{k_{\mathrm{ON}- Mb}}{\to}\\ {}\underset{k_{\mathrm{OFF}- Mb}}{\leftarrow}\end{array}}\ O-{M}_B $$

$$ O-{M}_A+{PS}_A\ {\displaystyle \begin{array}{c}\overset{k_{\mathrm{ON}- PS}}{\to}\\ {}\underset{k_{\mathrm{OFF}- PS}}{\leftarrow}\end{array}}\ O(PS) $$

$$ O-{M}_B+{PS}_B{\displaystyle \begin{array}{c}\overset{k_{\mathrm{ON}- PS}}{\to}\\ {}\underset{k_{\mathrm{OFF}- PS}}{\leftarrow}\end{array}}\ O(PS) $$

$$ O-{M}_A+ PI4{P}_A{\displaystyle \begin{array}{c}\overset{k_{\mathrm{ON}- PI4P}}{\to}\\ {}\underset{k_{\mathrm{OFF}- PI4P}}{\leftarrow}\end{array}}\ O(PI4P) $$

$$ O-{M}_B+ PI4{P}_B{\displaystyle \begin{array}{c}\overset{k_{\mathrm{ON}- PI4P}}{\to}\\ {}\underset{k_{\mathrm{OFF}- PI4P}}{\leftarrow}\end{array}}\ O(PI4P) $$

*O* corresponds to an empty form of the ORD in solution. *O* − *M*_A_ and *O* − *M*_B_ correspond to ORD in an empty state, bound to A and B membrane, respectively. *O*(PS) and *O*(PI4P) correspond to ORD in a soluble state in 1:1 complex with a PS and PI(4)P molecule, respectively. PS_A_ and PS_B_ correspond to the PS pool in the A and B membrane, respectively. PI4P_A_ and PI4P_B_ are the PI(4)P pools in the A and B membrane, respectively. The time evolution of the PS and PI(4)P concentrations in A and B membranes respectively was determined by integrating a system of ordinary differential equations corresponding to our model. PS transfer was simulated in non-exchange conditions by considering [PS_A_] = 5 μM, [PS_B_] = 0 μM, [PI4P_A_] = [PI4P_B_] = 0 μM, and [O] = 200 nM at *t* = 0. The concentrations of the other forms of the ORD were considered to be equal to 0. PS transfer in exchange conditions was calculated considering that [PI4P_B_] was initially equal to 5 μM. Inversely, PI(4)P transfer was simulated in non-exchange conditions considering [PI4P_B_] = 5 μM, [PI4P_A_] = 0 μM, and [PS_A_] = [PS_B_] = 0 μM. In exchange conditions, [PS_A_] value was set at 5 μM. All *k*_ON-Mb_ and *k*_OFF-Mb_ rates were set to 10 µM^-1^ s^−1^ and 1 s^−1^, respectively. All *k*_ON-lipid_ and *k*_OFF-lipid_ rates were set to 10 μM^−1^ s^−1^ and 1 s^−1^, respectively, unless otherwise specified. The implementation of the kinetic model and the simulations were carried out with the software GEPASI v3.3 [64].

### Cell culture, transfection, and drug treatment

HeLa cells (Hela W.S, STRB7753, ATCC, tested for mycoplasma contamination) were grown in Dulbecco’s modified Eagle’s medium (DMEM) supplemented with 10% (v/v) fetal bovine serum (Eurobio) at 37 °C under 5% CO_2._ Cells were seeded (40,000 cells per condition) in an 8-well coverslip (Ibidi). The next day, the cells were transiently transfected with 125 ng of GFP-C2_Lact_ (Addgene, #22852) plasmid only or additionally with 125 ng of Lyn11-FRB-mCherry plasmid (Addgene, #38004) using Lipofectamine 3000 (Thermo Fisher Scientific) according to the manufacturer’s instructions. To deplete cholesterol from the PM, cells were treated for 24 h with 2.5 μg/mL of U18666A (Sigma) following the transfection step.

### Microscopy and image analysis

One day after transfection, the cells were observed in live conditions using a wide-field microscope (Olympus IX83, × 60) or a confocal microscope (Zeiss LSM780, × 63). Prior to the observation, the medium was replaced by HEPES-containing DMEM devoid of red phenol. The depletion of cholesterol in the PM was assessed in cells that were only transfected by GFP-C2_Lact_ using the fluorescent sterol sensor mCherry-D4-His_6_. These cells were incubated for 10 min at room temperature with the protein added at 1:500 in DMEM. Then the cells were rinsed twice with HEPES-containing DMEM devoid of red phenol and the cells were immediately observed. To quantify the recruitment of GFP-C2_Lact_ to the PM, line scan analyses of a large set of cells were performed using Fiji ImageJ 2.1.0 [65]. A line was manually drawn across each individual cell and the peak intensity at the PM region was normalized with the intensity of the cytosolic region and then plotted for quantification. The localization of the PM was ascertained for each measurement by using Lyn11-FRB-mCherry as an internal reference.

### Statistical analyses

Statistical analyses were performed using an unpaired Mann–Whitney non-parametric *U* tests (Prism, GraphPad). *p* values < 0.05, < 0.01, and < 0.0001 are identified with 1, 2, and 4 asterisks, respectively. ns: *p* ≥ 0.05. The number of replicates (*n*) used for calculating statistics is specified in the figure legends.

## Supplementary Information

**Additional file 1.**

**Additional file 2.**

## Data Availability

All data generated or analyzed during this study are included in this published article and its supplementary information files.

## Abbreviations

C2_Lact_: C2 domain of lactadherin
DOPC: 1,2-Dioleoyl-*sn*-glycero-3-phosphocholine
ER: Endoplasmic reticulum
FRB: FKBP-rapamycin-binding
FRET: Fluorescence Resonance Energy Transfer
LTPs: Lipid transfer proteins
NBD: Nitrobenzoxadiazole
ORD: OSBP-related domain
ORP: Oxysterol-binding protein-related proteins
Osh: Oxysterol-binding homology
PH: Pleckstrin homology
PH_FAPP_: PH domain of the four-phosphate-adaptor protein
PI: Phosphatidylinositol
PI(4)P: Phosphatidylinositol 4-phosphate
PI(4,5)P_2_: Phosphatidylinositol 4,5-bisphosphate
PIPs: Polyphosphoinositides
PS: Phosphatidylserine
PM: Plasma membrane
Rhod-PE: 1,2-Dipalmitoyl-*sn*-glycero-3-phosphoethanolamine-N-(lissamine rhodamine B sulfonyl
TSA: Thermal shift assay
