# Supplementary material for: Functional analyses of phosphatidylserine/PI(4)P exchangers with diverse lipid species and membrane contexts reveal unanticipated rules on lipid transfer
Source: BMC Biol. 2021 Nov 20;19:248. doi: 10.1186/s12915-021-01183-1 (PMC8606082; doi:10.1186/s12915-021-01183-1)
Supplement: Supplementary file 1 — Additional file 1. [file 12915_2021_1183_MOESM1_ESM.pdf]

Additional file for

Functional analyses of phosphatidylserine/PI(4)P exchangers with diverse lipid species and membrane contexts reveal unanticipated rules on lipid transfer

Souade Ikhlef[1]*, Nicolas-Frédéric Lipp[1,3]*, Vanessa Delfosse[2], Nicolas Fuggetta[1], William Bourguet[2], Maud Magdeleine[1] and Guillaume Drin[1]

Correspondence to: drin@ipmc.cnrs.fr

Figures S1 to S9

Fig. S1. Kinetics of PS transfer measured with Osh6p and ORD8 with different PS species and L_B liposomes containing or not PI(4)P

Fig. S2. Kinetics of PI(4)P transfer by Osh6p and ORD8 in the context of PS/PI(4)P exchange depends on the length and unsaturation degree of PS species

Fig. S3. Transfer kinetics of 16:0/18:1-PS and 18:0/18:1-PS and PI(4)P with different unsaturation degree, measured with Osh6p or ORD8 in non-exchange and exchange conditions with different PS and PI(4)P combinations

Fig. S4. FRET between Osh6p or ORD8 and NBD-PS

Fig. S5. Relationship between the affinity of ORD8 for PS and PI(4)P species and its ability to transfer them

Fig. S6. Analysis of the relationship between ORD's affinity for PS and PI(4)P and its ability to transfer these lipids between membranes

Fig. S7. Biochemical characterization of NBD-$PH_{PLC\delta1}$

Fig. S8. PIP selectivity of Osh6p and ORD8

Fig. S9. Impact of sterol on PS/PI(4)P exchange

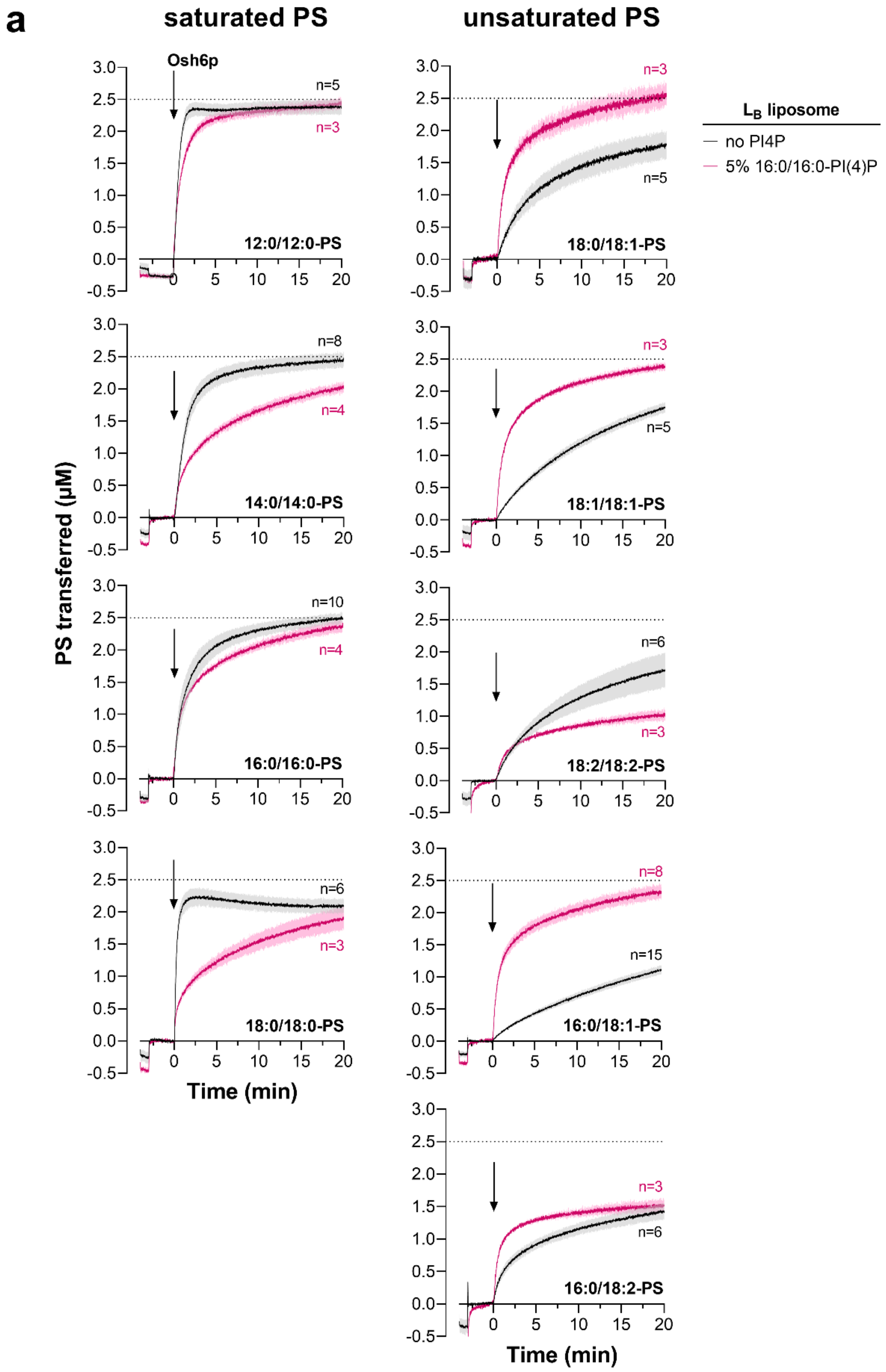

Fig. S1. (see legend on next page)

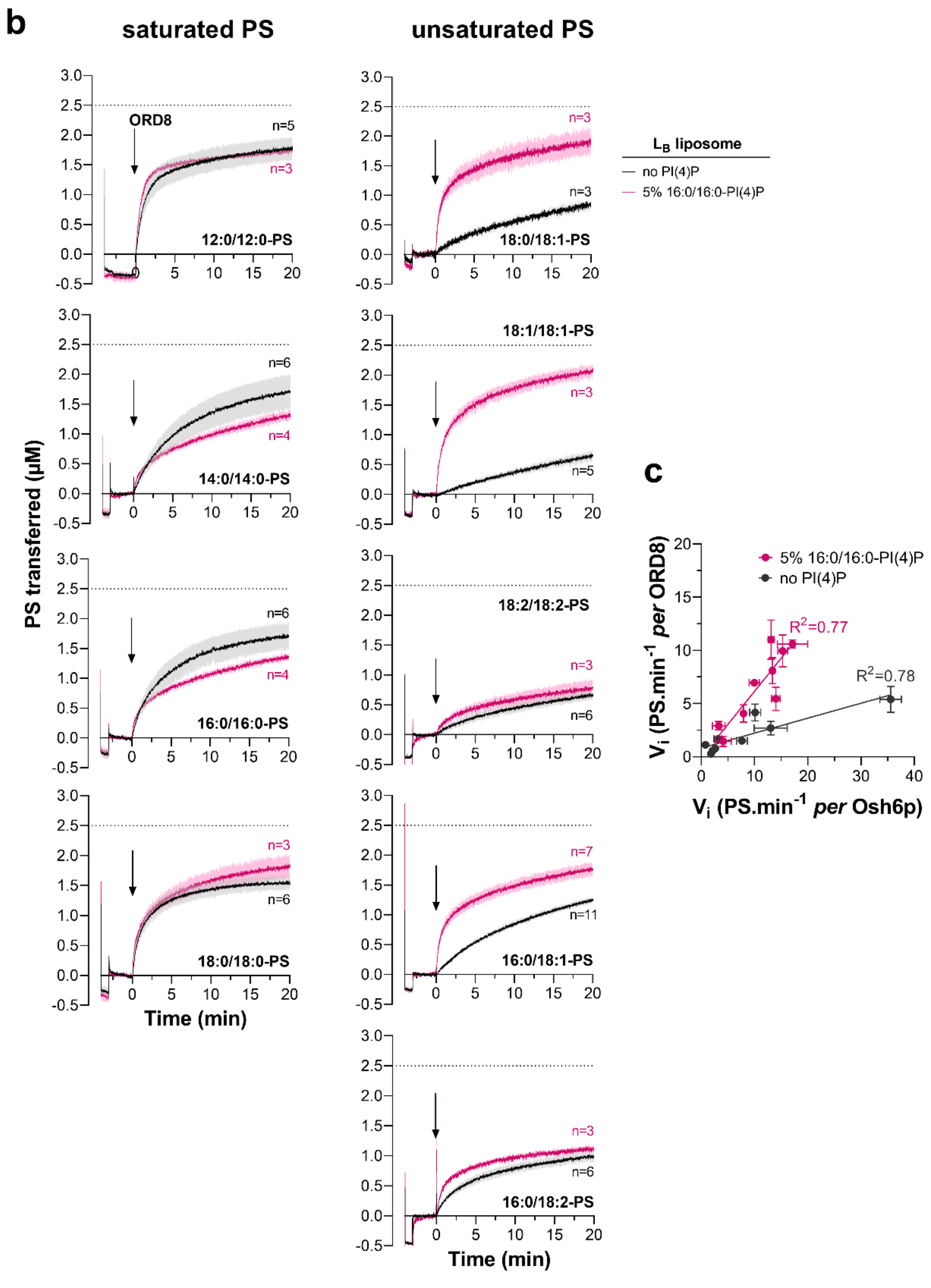

Fig. S1. Kinetics of PS transfer measured with Osh6p and ORD8 with different PS species and L_B liposomes containing or not PI(4)P. (a) DOPC liposomes (200 µM total lipid, L_A) containing 2% Rhod-PE and 5% PS were mixed with NBD-C2$_{Lact}$ (250 nM) at 30°C. After one minute, DOPC liposomes (200 µM lipids, L_B) containing or not 5% 16:0/16:0-PI(4)P were added. After 3 min, Osh6p (200 nM) was injected. Dark trace: without PI(4)P, pink trace: with PI(4)P. Each trace is the mean ± s.e.m. of kinetics recorded in independent experiments (n = 3-15). A dashed line indicates the level of PS in L_B liposomes that should be observed if the liposomes are fully equilibrated. **(b)** Similar experiments were carried out with ORD8 (240 nM) at 37°C. Each curve is the mean ± s.e.m. of curves recorded in independent experiments (n = 3-11). **(c)** Initial PS transfer rates measured with Osh6p *vs* those measured with ORD8 in the presence of L_B liposomes enriched or not with 5% 16:0/16:0-PI(4)P.

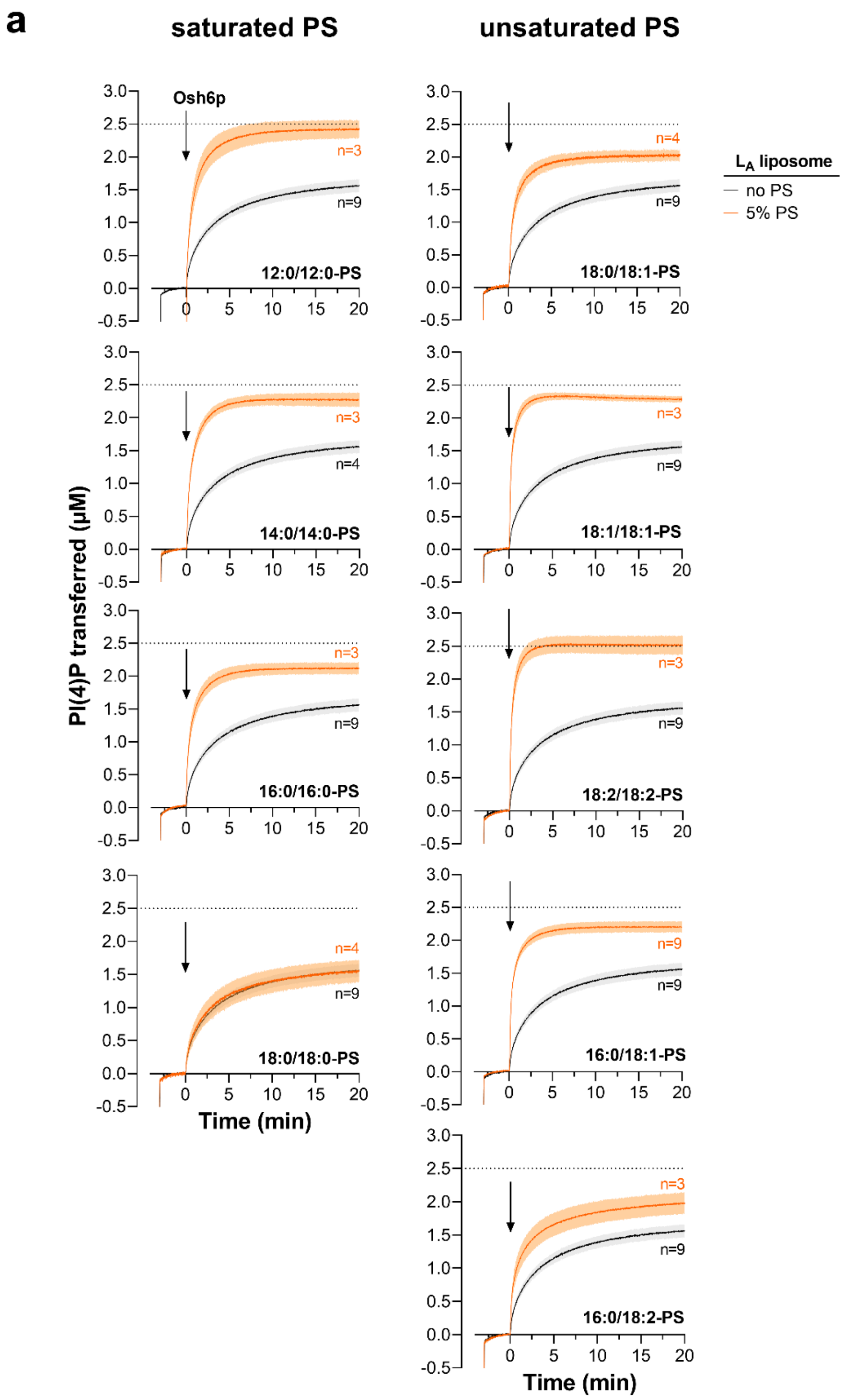

Fig. S2. (see legend on next page)

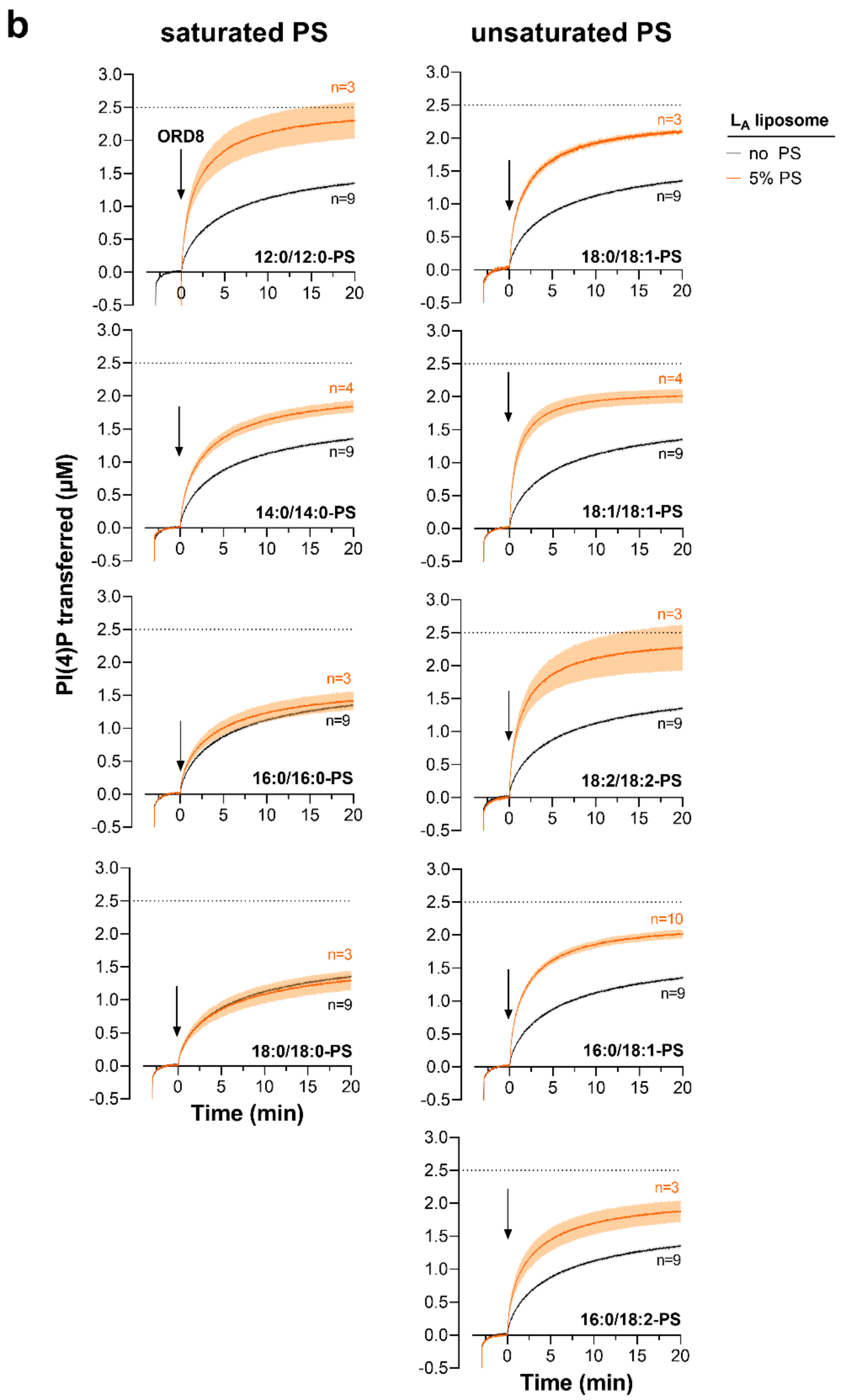

Fig. S2. Kinetics of PI(4)P transfer by Osh6p and ORD8 in the context of PS/PI(4)P exchange depends on the length and unsaturation degree of PS species. (a) DOPC liposomes (200 µM lipids, L_B) containing 5% 16:0/16:0-PI(4)P were mixed with NBD-PH_{FAPP} (250 nM) at 30°C. After one minute, DOPC liposomes (200 µM total lipid, L_A) containing 2% Rhod-PE and 5% PS were added. After 3 min, Osh6p (200 nM) was injected. Dark trace: without PS, orange trace: with PS. Each trace is the mean ± s.e.m. of independent kinetics (n = 3-9). **(b)** Similar experiments were performed with ORD8 (240 nM) at 37°C. Each curve is the mean ± s.e.m. of independent kinetics traces (n = 3-10). A dashed line indicates the PI(4)P amount that should be transferred in L_B liposomes at equilibrium.

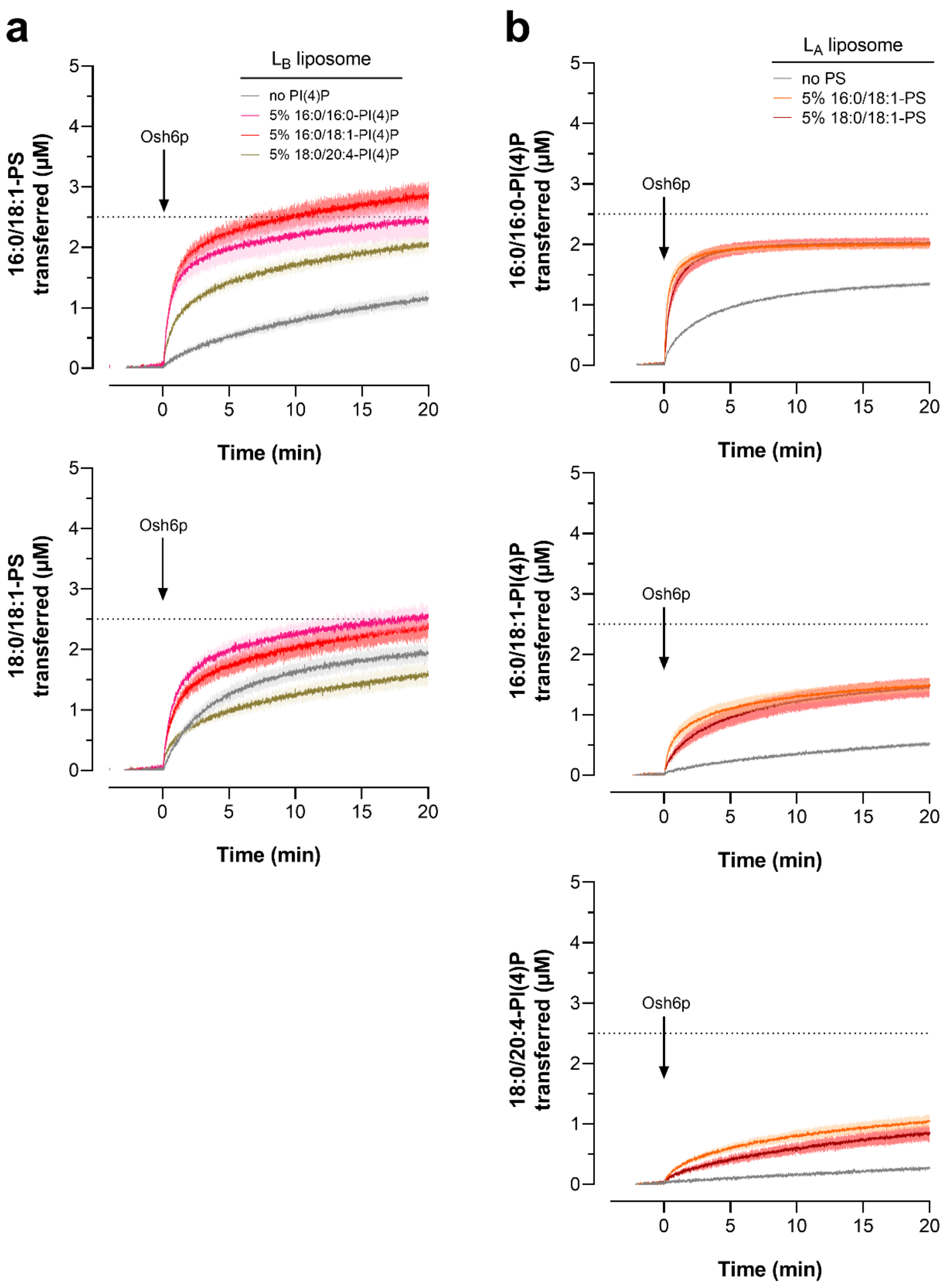

Fig. S3. (see legend on next page)

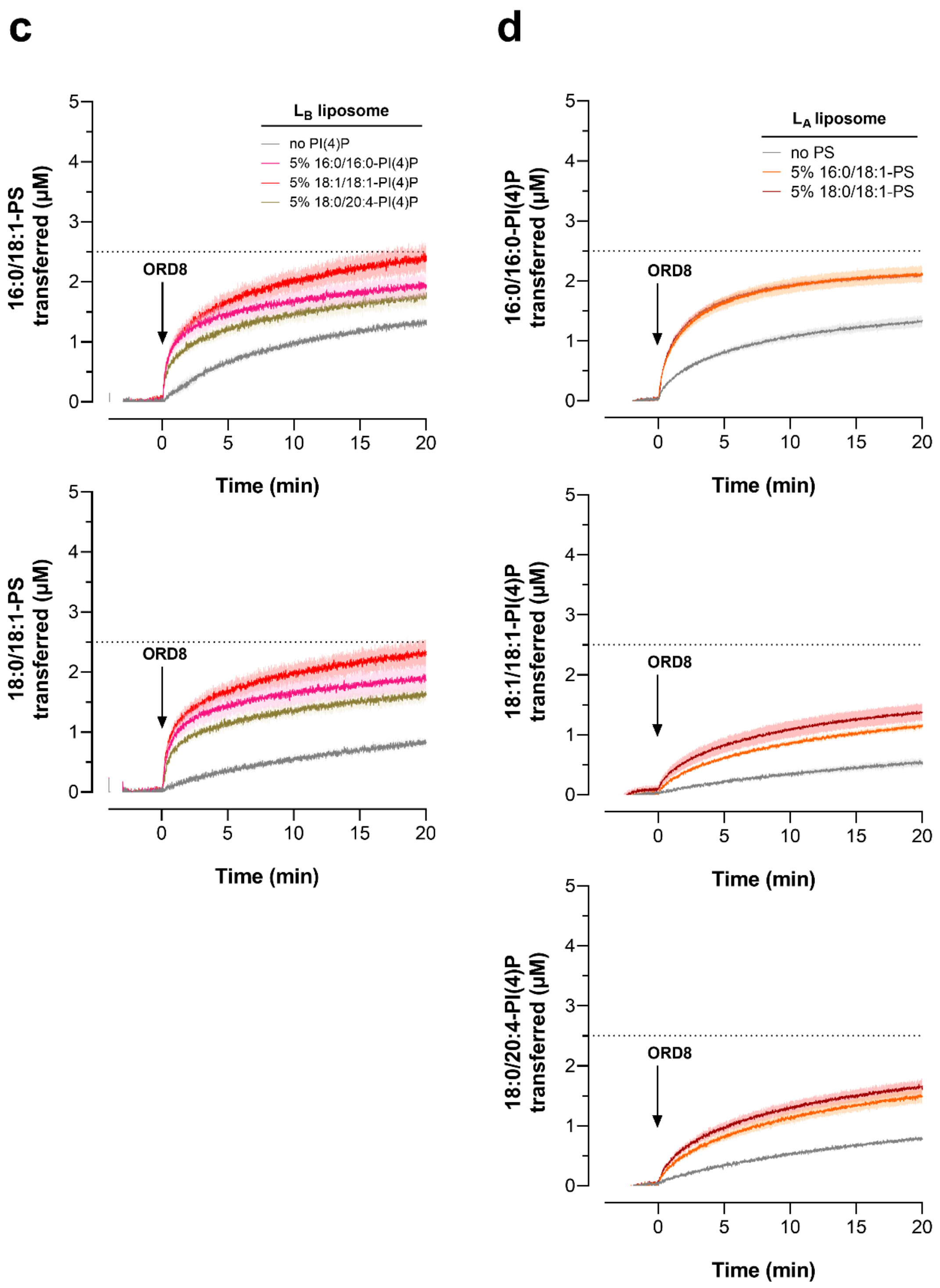

Fig. S3. Transfer kinetics of 16:0/18:1-PS and 18:0/18:1-PS and PI(4)P with different unsaturation degree, measured with Osh6p or ORD8 in non-exchange and exchange conditions with different PS and PI(4)P combinations. (a) DOPC liposomes (200 µM total lipid, L_A) containing 2% Rhod-PE and 5% 16:0/18:1-PS or 18:0/18:1-PS were mixed with NBD-C2$_{Lact}$. After one minute, DOPC liposomes (200 µM lipids, L_B) containing or not 5% of a particular PI(4)P species were added. After 3 min, Osh6p was injected. **(b)** L_B liposomes (200 µM lipids) containing a given PI(4)P species (at 5%) were pre-incubated with NBD-PH$_{FAPP}$. Next, L_A liposomes (200 µM total lipid) containing 2% Rhod-PE, enriched or not with 5% 16:0/18:1-PS or 18:0/18:1-PS, and Osh6p were sequentially added. Experiences were conducted at 30°C with 200 nM Osh6p.**(c)** Measurements were taken as in **(a)** but with 240 nM ORD8 at 37°C and using 18:1/18:1-PI(4)P instead of 16:0/18:1-PI(4)P. **(d)** Same experiments as in **(b)** but performed with ORD8 and by replacing 16:0/18:1-PI(4)P by 18:1/18:1-PI(4)P. Each trace is the mean ± s.e.m. of independent kinetics (n = 3-4).

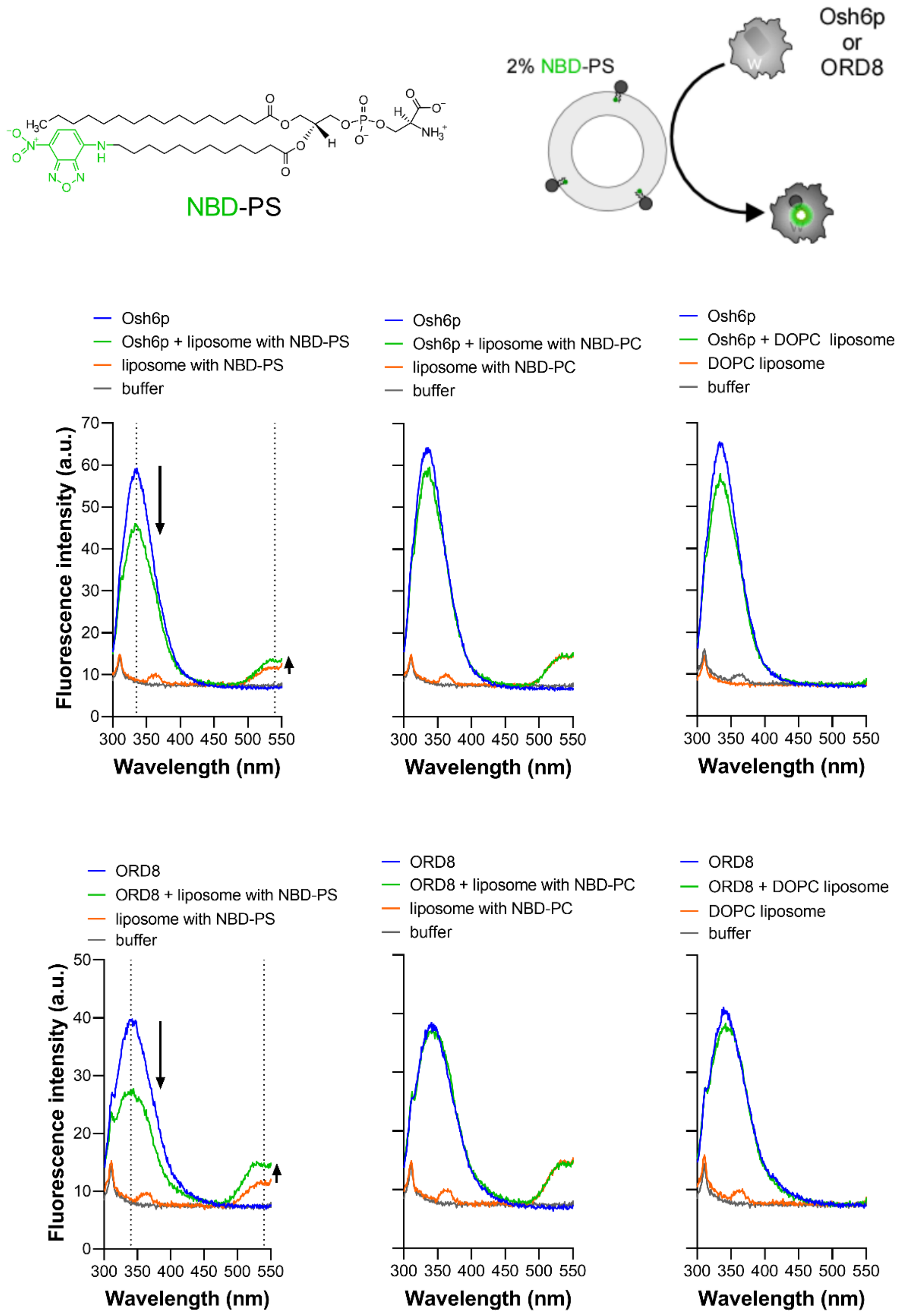

Fig. S4. FRET between Osh6p or ORD8 and NBD-PS.
Emission spectra of Osh6p and ORD8 (240 nM) alone in HK buffer or in the presence of liposomes (100 µM total lipid) containing either 2% NBD-PS or NBD-PC, measured at 30°C upon excitation at 280 nm. A control experiment was done with pure DOPC liposomes. For each condition, a spectrum was recorded in the absence of protein. Arrows indicate the diminution of the Trp fluorescence and the increase in NBD fluorescence that were observed once liposomes doped with NBD-PS were added to Osh6p or ORD8, indicative of a FRET process between each protein and the fluorescent lipid.

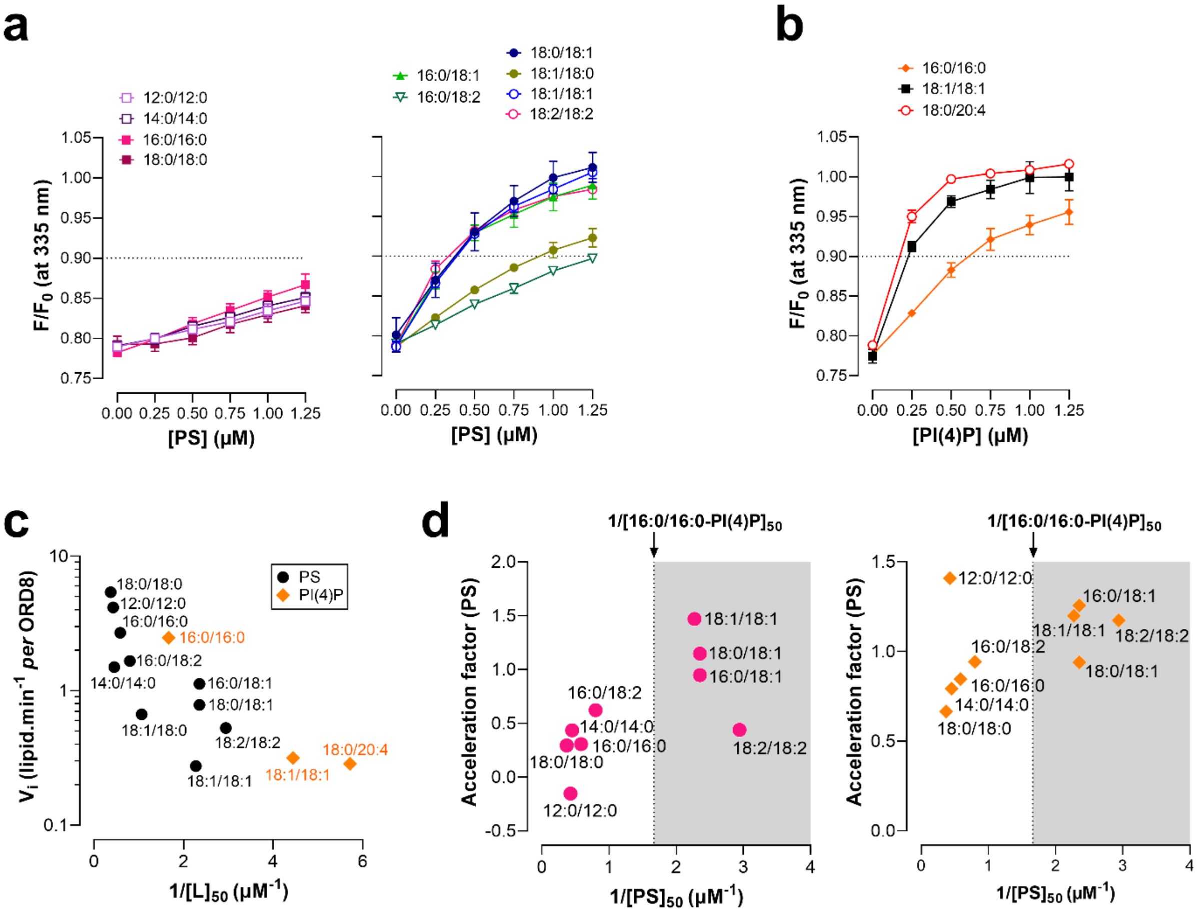

Fig. S5. Relationship between the affinity of ORD8 for PS and PI(4)P species and its ability to transfer them.

(a) Competition assays. DOPC liposomes (100 µM total lipid, final concentration) doped with 2% NBD-PS, were added to ORD8 (240 nM) in HK buffer at 30°C. The sample was excited at 280 nm and the emission was measured at 335 nm. Incremental amounts of liposome, containing a given PS species at 5%, were injected to the sample. The fluorescence was normalized considering the initial F_{max} fluorescence, prior to the addition of NBD-PS-containing liposomes, and the dilution effect due to liposomes addition. Data are represented as mean ± s.e.m. (n = 3). **(b)** Same experiments as in **(a)** except that liposomes containing a given PI(4)P species at 5% were injected. Data are represented as mean ± s.e.m. (n = 4 with 16:0/16:0-PI(4)P, n = 3 for other PI(4)P species). **(c)** Initial transfer rates determined under non-exchange conditions for PS and PI(4)P subspecies with ORD8 (shown in Figure 1c, d and 2a) as a function of $1/[L]_{50}$ values (right panel) determined for each lipid subspecies. **(d)** Acceleration factors determined from PS and 16:0/16:0-PI(4)P transfer rates obtained in the experiments shown in Figure 1b and c, as a function of the $1/[L]_{50}$ value determined for each PS subspecies.

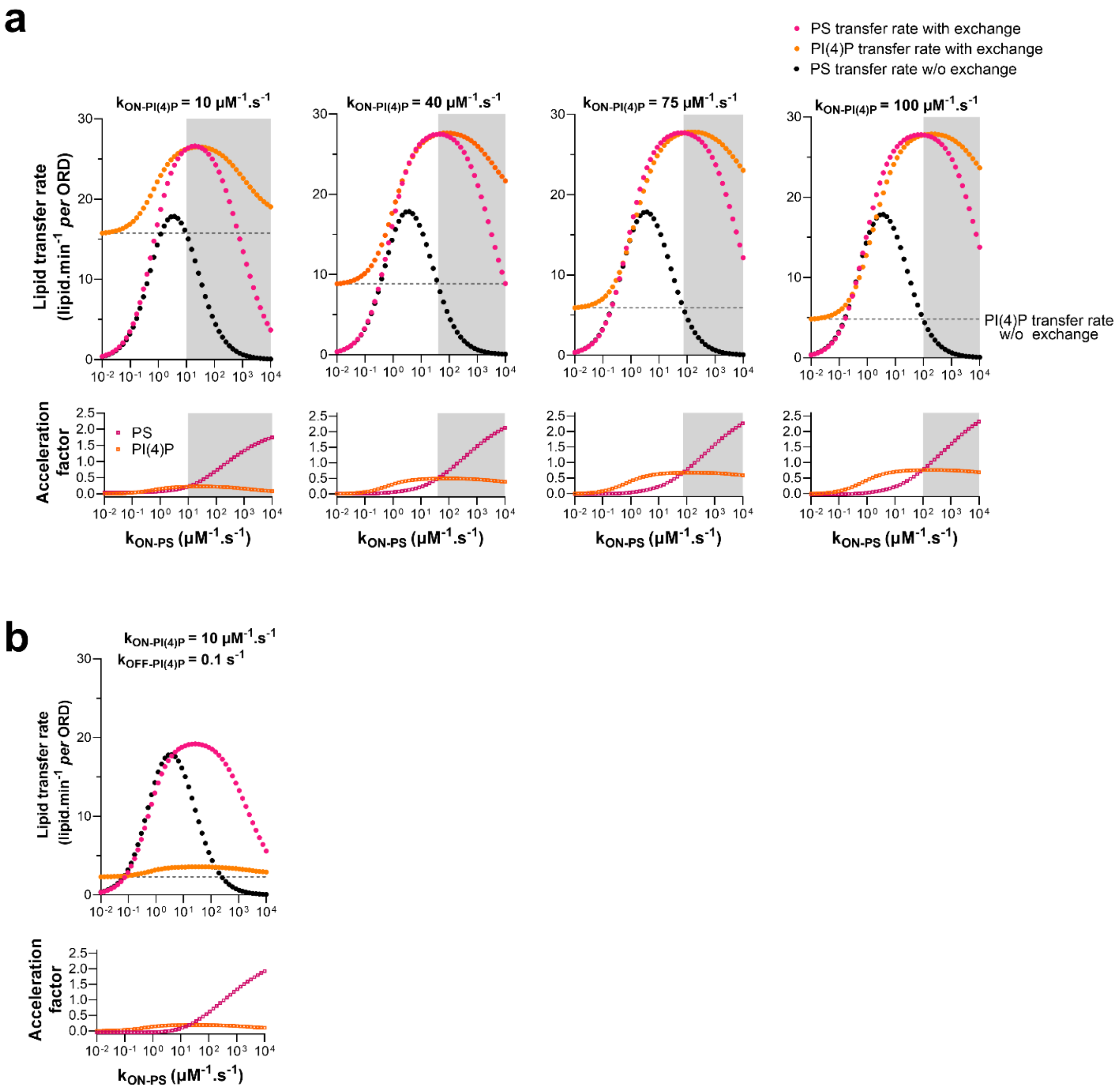

Fig. S6. Analysis of the relationship between ORD's affinity for PS and PI(4)P and its ability to transfer these lipids between membranes.
(a) Initial PS (pink dots) and PI(4)P transfer rates (orange dots) were calculated as a function of k_{ON-PS} for different $k_{ON-PI(4)P}$ values (10 $\mu M^{-1}.s^{-1}$, as shown in Figure 5, but also 40, 75 and 100 $\mu M^{-1}.s^{-1}$), considering that PS and PI(4)P were initially present at 5% in the A and B membranes, respectively (exchange condition). The PS transfer rates, calculated as a function of k_{ON-PS} values, and the PI(4)P transfer rate under non-exchange condition are shown (black dots and dashed line, respectively). The grey areas correspond to regimes where $k_{ON-PS} > k_{ON-PI(4)P}$ *i.e.*, the ORD has more affinity for PS than PI(4)P. The acceleration factors, calculated for PS and PI(4)P are shown. **(b)** Simulations were carried out as in **(a)** with $k_{ON-PI(4)P}$ = 10 $\mu M^{-1}.s^{-1}$ and $k_{OFF-PI(4)P}$ = 0.1 s^{-1}. Acceleration factors are shown.

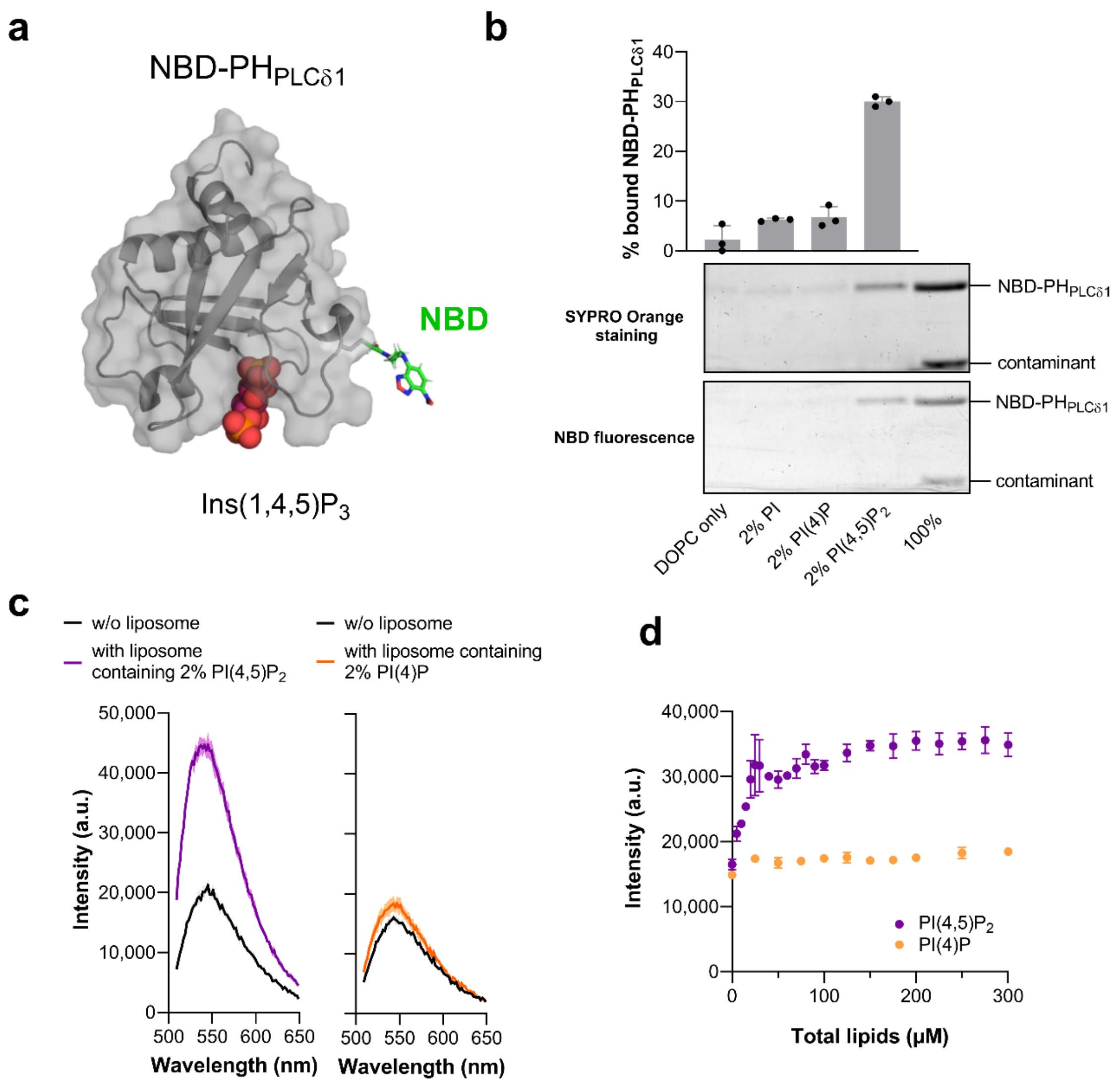

Fig. S7. Biochemical characterization of NBD-PH$_{PLCδ1}$.

(a) Tridimensional model of the NBD-labeled PH$_{PLCδ1}$ based on the crystal structure of the PH domain of the *Rattus norvegicus* Phospholipase C δ1 (PDB ID: 1MAI[1]). The solvent-exposed cysteine C48 was mutated into a serine; serine S61 was replaced by a cysteine. An N,N'-dimethyl-N-(thioacetyl)-N'-(7-nitrobenz-2-oxa-1,3-diazol-4-yl)ethylenediamine moiety (in stick, with carbon in green, nitrogen in blue and oxygen in red), built manually and energetically minimized, is grafted to the thiol function of C61. The inositol 1,4,5-trisphosphate (Ins(1,4,5)P$_3$) molecule, corresponding to the polar head of PI(4,5)P$_2$ is represented in sphere mode with oxygen in red and phosphate in orange. The figure was prepared with PyMOL (http://pymol.org/). **(b)** Flotation assays. NBD-PH$_{PLCδ1}$ (1 µM) was incubated in HK buffer at room temperature for 10 min under agitation with liposomes (1.5 mM total lipid concentration) only made of DOPC or containing 2% liver PI, 2% 18:0/20:4-PI(4)P or 2% 18:0/20:4-PI(4,5)P$_2$. After centrifugation, the liposomes were recovered at the top of sucrose cushions and analyzed by SDS-PAGE. The presence of NBD-PH$_{PLCδ1}$ in the top fraction (lane 1 to 4) and in the reference lane (lane 5) was visualized by direct fluorescence, and after staining the gel with SYPRO Orange, using a fluorescence imaging system. The amount of protein recovered in the top fraction was quantified based on the SYPRO Orange signal and the percentage of NBD-PH$_{PLCδ1}$ bound to liposomes was determined using the content of lane 5 (100%) as a reference. Data are represented as mean ± s.e.m. (n = 3). **(c)** Fluorescence spectra of NBD-PH$_{PLCδ1}$ (250 nM) in buffer alone or mixed with DOPC liposome (350 µM) containing either 2% PI(4)P or 2% PI(4,5)P$_2$. **(d)** NBD intensity measured at 535 nm as function of total lipid concentration and with liposome composed of DOPC, DOPC/PI (98:2), DOPC/PI(4)P (98:2) or DOPC/PI(4,5)P$_2$ (98:2). Data are represented as mean ± s.e.m. (n = 3).

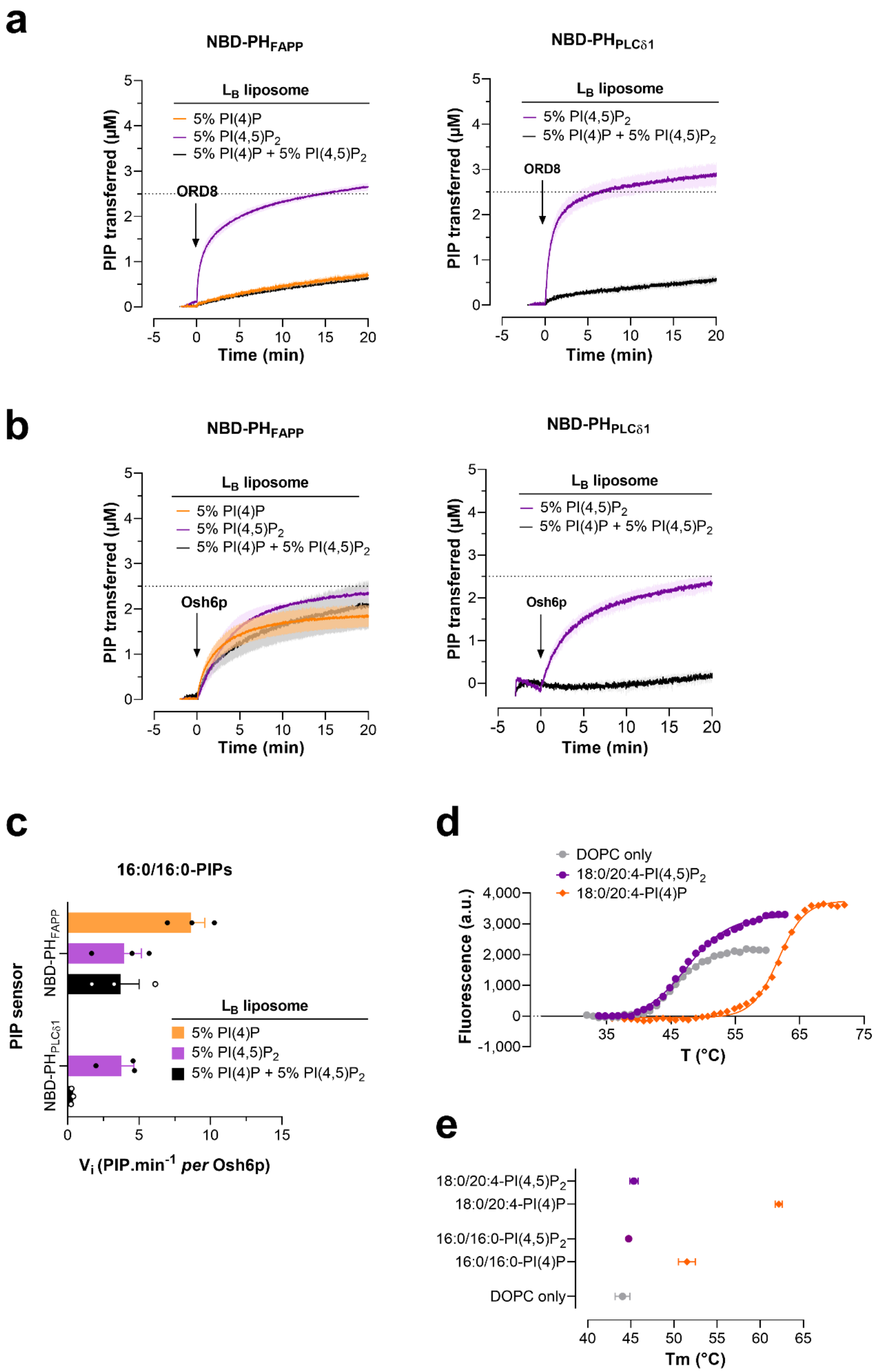

Fig. S8. (see legend on next page)

Fig. S8. PIP selectivity of ORP8 and Osh6p. (a) Intermembrane PI(4)P and PI(4,5)P_2 transfer activity of ORD8 or Osh6p when PI(4)P, PI(4,5)P_2 or both PIPs were present in donor L_B liposomes. Experiments were performed with ORD8 as for those shown in **Figure 6a** except that 18:0/20:4-PIPs were assayed. The injection of the LTP set the time to zero. Each trace is the mean ± s.e.m. of independent kinetics (n = 3). **(b)** Intermembrane PIP transfer activity of Osh6p. Experiments were performed at 30°C as described in **Figure 6a** using Osh6p (200 nM final concentration) and liposomes containing PI(4)P and PI(4,5)P_2 with 16:0/16:0 acyl chains. Each trace is the mean ± s.e.m. of independent kinetics (n = 3). **(c)** Initial transfer rates of 16:0/16:0-PIPs determined with NBD-PH_{FAPP} and NBD-$PH_{PLC\delta1}$ based on the kinetic curves measured with Osh6p and shown in panel **(b)**. Data are represented as mean ± s.e.m. (error bars, n = 3). **(d)** Melting curves of Osh6p loaded with 18:0/20:4-PI(4)P or 18:0/20:4-PI(4,5)P_2. A control experiment with Osh6p incubated with DOPC liposomes without ligands is shown. **(e)** Melting temperatures (T_m) determined for Osh6p incubated with liposome devoid of lipid ligand (DOPC only) or loaded with PI(4)P or PI(4,5)P_2 with 16:0/16:0 or 18:0/20:4 composition. Data are represented as mean ± s.e.m (n = 3) except for 18:0/20:4-PI(4)P and 16:0/16:0-PI(4,5)P_2 (n = 2).

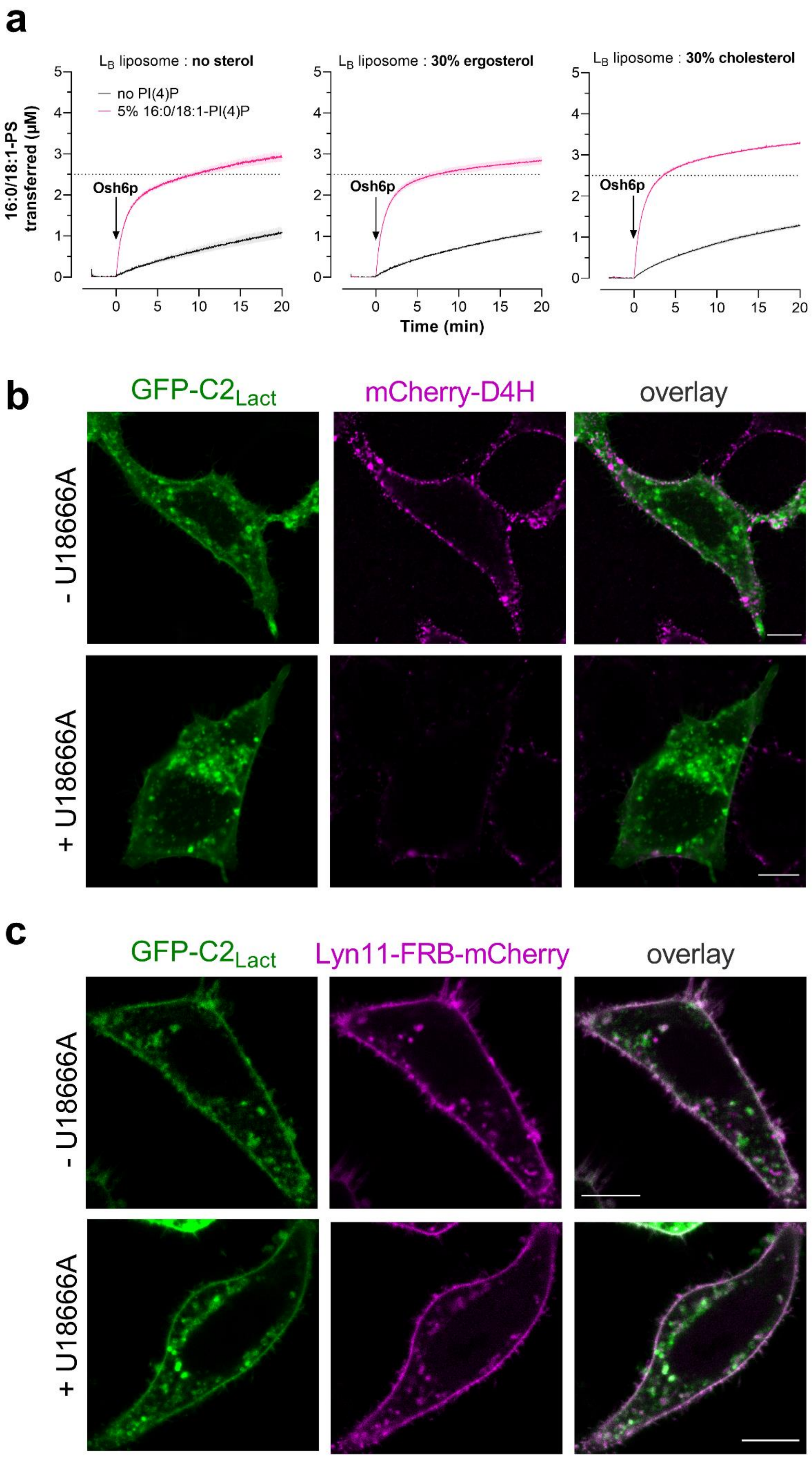

Fig. S9. (see legend on next page)

Fig. S9. Impact of sterol on PS/PI(4)P exchange.
(a) Transfer kinetics of 16:0/18:1-PS from L_A liposomes (200 µM total lipid, 5% PS) to L_B liposomes (200 µM) without or with 30% sterol (cholesterol or ergosterol), doped or not with 5% 16:0/18:1-PI(4)P (at the expense of PC). Measurements were done at 30°C with Osh6p (200 nM). Each trace is the mean ± s.e.m. of kinetics recorded in independent experiments (n = 4-5). **(b)** Confocal images of live GFP-$C2_{Lact}$-expressing HeLa cells (green) treated or not with U18666A for 24 h at 37°C. Cholesterol in the PM was detected by incubating the cells for 10 min with mCherry-D4H (purple) at room temperature and then washed with medium prior to imaging. The overlay panel shows merged green and magenta images. Scale bars: 10 µm. **(c)** Confocal images of live cells that co-express GFP-$C2_{Lact}$ (green) and Lyn11-FRB-mCherry (magenta) and treated or not with U18666A for 24 h at 37°C. The treatment with the drug did not change the localization of Lyn11-FRB-mCherry used as a stable marker of the PM. The overlay panel shows merged green and magenta images. Scale bars: 10 µm.

References

1. Ferguson KM, Lemmon MA, Schlessinger J, Sigler PB: **Structure of the high affinity complex of inositol trisphosphate with a phospholipase C pleckstrin homology domain**. *Cell* 1995, **83**(6):1037-1046.
