# Supplementary material for: Functional analyses of phosphatidylserine/PI(4)P exchangers with diverse lipid species and membrane contexts reveal unanticipated rules on lipid transfer
Source: BMC Biol. 2021 Nov 20;19:248. doi: 10.1186/s12915-021-01183-1 (PMC8606082; doi:10.1186/s12915-021-01183-1)
Supplement: Supplementary file 2 — Additional file 2. [file 12915_2021_1183_MOESM2_ESM.pdf]

Additional file for

Functional analyses of phosphatidylserine/PI(4)P exchangers with diverse lipid species and membrane contexts reveal unanticipated rules on lipid transfer

Souade Ikhlef[1]*, Nicolas-Frédéric Lipp[1,3]*, Vanessa Delfosse[2], Nicolas Fuggetta[1], William Bourguet[2], Maud Magdeleine[1] and Guillaume Drin[1]

Correspondence to: drin@ipmc.cnrs.fr

Table S1 to S9

Table S1 – Individual data points (F/F_0 values) related to Fig. 4b

Table S2 - Individual data points (F/F_0) related to Fig. 4c

Table S3 - Individual data points (Tm) related to Fig. 4e

Table S4 – Individual data points (F/F_0) related to Fig. 6f

Table S5 – Individual data points (F/F_0) related to Fig. S5a

Table S6 – Individual data points (F/F_0) related to Fig. S5b

Table S7 - Individual data points (fluo) related to Fig. S7d

Table S8 - Individual data points (Tm) related to Fig. S8e

Table S1 – Individual data points (F/F_0 values) related to Fig. 4b

[PS]	F/F_0 (335nm)														
(µM)	12:0/12:0-PS			14:0/14:0-PS			16:0/16:0-PS			18:0/18:0-PS			18:0/18:1-PS		
	n1	n2	n3	n1	n2	n3	n1	n2	n3	n1	n2	n3	n1	n2	n3
0	0.804	0.802	0.805	0.822	0.801	0.820	0.798	0.788	0.829	0.798	0.826	0.783	0.800	0.814	0.797
0.25	0.836	0.844	0.844	0.866	0.879	0.903	0.881	0.889	0.971	0.813	0.841	0.790	0.901	0.962	0.936
0.5	0.858	0.875	0.872	0.896	0.921	0.964	0.912	0.924	1.025	0.818	0.842	0.797	0.921	0.999	0.974
0.75	0.872	0.882	0.897	0.910	0.950	1.000	0.934	0.949	1.045	0.824	0.851	0.799	0.930	1.010	0.980
1	0.886	0.899	0.913	0.911	0.967	1.021	0.950	0.954	1.053	0.834	0.846	0.797	0.932	1.017	0.987
1.25	0.892	0.896	0.933	0.916	0.965	1.041	0.957	0.955	1.052	0.836	0.846	0.803	0.937	1.027	0.981

	F/F_0 (335nm															
[PS]	18:1/18:0-PS			18:1/18:1-PS			18:2/18:2-PS				16:0/18:1-PS			16:0/18:2-PS		
(µM)	n1	n2	n3	n1	n2	n3	n1	n2	n3	n4	n1	n2	n3	n1	n2	n3
0	0.811	0.811	0.815	0.804	0.796	0.805	0.799	0.807	0.811	0.820	0.805	0.801	0.791	0.801	0.807	0.821
0.25	0.859	0.878	0.885	0.927	0.968	0.979	0.900	0.927	0.948	0.942	0.949	1.006	0.990	0.817	0.847	0.851
0.5	0.884	0.926	0.924	0.949	0.988	1.021	0.913	0.964	0.972	0.971	0.962	1.030	1.008	0.836	0.865	0.890
0.75	0.909	0.957	0.955	0.952	1.003	1.025	0.918	0.980	0.994	0.983	0.967	1.035	1.005	0.852	0.877	0.909
1	0.920	0.977	0.963	0.959	1.009	1.029	0.916	0.985	0.994	0.989	0.968	1.033	1.006	0.864	0.896	0.937
1.25	0.934	0.996	0.969	0.966	1.025	1.032	0.915	0.998	0.999	0.993	0.967	1.045	1.006	0.876	0.890	0.942

Table S2 - Individual data points (F/F_0) related to Fig. 4c

	F/F_0 (335nm)									
[PI(4)P]	16:0/16:0-PI(4)P			16:0/18:1-PI(4)P				18:0/20:4-PI(4)P		
(µM)	n1	n2	n3	n1	n2	n3	n4	n1	n2	n3
0	0.773	0.774	0.851	0.786	0.782	0.854	0.822	0.784	0.819	0.839
0.25	0.844	0.867	0.908	0.945	0.954	0.962	0.932	0.947	0.934	0.960
0.5	0.910	0.928	0.960	0.969	0.963	0.960	0.945	0.966	0.937	0.971
0.75	0.935	0.949	0.981	0.973	0.970	0.961	0.943	0.971	0.937	0.975
1	0.945	0.948	0.987	0.975	0.979	0.963	0.947	0.975	0.939	0.976
1.25	0.942	0.945	0.988	0.989	0.981	0.964	0.939	0.980	0.939	0.986

Table S3 - Individual data points (Tm) related to Fig. 4e

	Tm (°C)			
Lipid subspecies	n1	n2	n3	n4
12:0/12:0-PS	44.62	47.27	46.05	
14:0/14:0-PS	45.34	47.12	45.73	
16:0/16:0-PS	46.30	46.72	49.26	
18:0/18:0-PS	44.15	44.35	46.41	
18:0/18:1-PS	46.38	47.24	50.88	51.39
18:1/18:0-PS	48.13	50.13	48.61	
18:1/18:1-PS	47.01	46.86	50.31	51.45
18:2/18:2-PS	47.39	48.04	49.03	
16:0/18:1-PS	48.64	48.77	52.86	
16:0/18:2-PS	47.96	46.98	48.55	
16:0/16:0-PI(4)P	52.43	53.45	50.39	
16:0/18:1-PI(4)P	55.97	58.40	53.91	
18:0/20:4-PI(4)P	62.15	61.72	62.55	
DOPC only	45.57	46.82	45.73	43.19

Table S4 – Individual data points (F/F_0) related to Fig. 6f

[PIP]	F/F_0 (335nm) Osh6p											
	16:0/16:0-PI(4)P			18:0/20:4-PI(4)P			16:0/16:0-PI(4,5)P_2			18:0/20:4-PI(4,5)P_2		
(μM)	n1	n2	n3	n1	n1	n1	n1	n2	n3	n1	n2	n3
0	0.798	0.782	0.854	0.780	0.780	0.780	0.827	0.832	0.816	0.780	0.849	0.827
0.25	0.877	0.858	0.911	0.816	0.816	0.816	0.835	0.847	0.828	0.816	0.878	0.862
0.5	0.946	0.916	0.950	0.841	0.841	0.841	0.834	0.851	0.836	0.841	0.890	0.886
0.75	0.977	0.941	0.971	0.855	0.855	0.855	0.833	0.858	0.840	0.855	0.897	0.900
1	0.979	0.949	0.975	0.863	0.863	0.863	0.837	0.867	0.844	0.863	0.898	0.909
1.25	0.987	0.954	0.984	0.867	0.867	0.867	0.842	0.870	0.840	0.867	0.897	0.918

[PIP]	F/F_0 (335nm) ORD8											
	16:0/16:0-PI(4)P			18:0/20:4-PI(4)P			16:0/16:0-PI(4,5)P_2			18:0/20:4-PI(4,5)P_2		
(μM)	n1	n2	n3	n1	n2	n3	n1	n2	n3	n1	n2	n3
0	0.770	0.760	0.794	0.831	0.753	0.779	0.790	0.781	0.785	0.780	0.766	0.760
0.25	0.829	0.812	0.848	1.013	0.920	0.917	0.810	0.795	0.790	0.791	0.776	0.765
0.5	0.876	0.862	0.918	1.079	0.977	0.978	0.827	0.805	0.807	0.800	0.783	0.765
0.75	0.901	0.899	0.959	1.092	0.991	0.996	0.839	0.826	0.820	0.807	0.789	0.782
1	0.922	0.928	0.996	1.099	0.998	1.010	0.852	0.831	0.839	0.815	0.801	0.785
1.25	0.942	0.944	1.011	1.101	0.996	1.015	0.866	0.858	0.851	0.818	0.813	0.795

Table S5 – Individual data points (F/F_0) related to Fig. S5a

[PS]	F/F_0 (335nm)											
	12:0/12:0-PS			14:0/14:0-PS			16:0/16:0-PS			18:0/18:0-PS		
(μM)	n1	n2	n3	n1	n2	n3	n1	n2	n3	n1	n2	n3
0	0.788	0.785	0.795	0.785	0.794	0.794	0.777	0.786	0.784	0.776	0.812	0.789
0.25	0.805	0.792	0.802	0.789	0.798	0.812	0.796	0.804	0.796	0.780	0.811	0.788
0.5	0.818	0.804	0.812	0.809	0.815	0.821	0.824	0.828	0.803	0.788	0.817	0.797
0.75	0.831	0.817	0.815	0.818	0.829	0.832	0.842	0.844	0.818	0.798	0.833	0.822
1	0.850	0.822	0.832	0.837	0.840	0.845	0.860	0.859	0.836	0.812	0.843	0.834
1.25	0.855	0.847	0.839	0.842	0.848	0.863	0.888	0.871	0.842	0.825	0.855	0.842

[PS]	F/F_0 (335nm)																	
	18:0/18:1-PS			18:1/18:0-PS			18:1/18:1-PS			18:2/18:2-PS			16:0/18:1-PS			16:0/18:2-PS		
(μM)	n1	n2	n3	n1	n2	n3	n1	n2	n3	n1	n2	n3	n1	n2	n3	n1	n2	n3
0	0.790	0.843	0.771	0.783	0.789	0.787	0.780	0.800	0.779	0.782	0.788	0.795	0.791	0.780	0.792	0.794	0.790	0.787
0.250	0.884	0.898	0.828	0.821	0.822	0.825	0.876	0.865	0.857	0.893	0.895	0.864	0.891	0.857	0.861	0.811	0.810	0.819
0.50	0.956	0.953	0.883	0.859	0.854	0.859	0.932	0.933	0.919	0.942	0.934	0.918	0.939	0.910	0.940	0.843	0.833	0.843
0.750	0.991	0.987	0.930	0.893	0.876	0.888	0.964	0.974	0.949	0.962	0.967	0.945	0.965	0.920	0.971	0.862	0.848	0.868
10	1.025	1.012	0.958	0.921	0.888	0.914	0.985	0.994	0.973	0.974	0.982	0.969	0.980	0.942	1.002	0.887	0.871	0.886
1.25	1.035	1.026	0.974	0.941	0.902	0.926	1.010	1.016	0.990	0.984	0.987	0.981	1.000	0.955	1.012	0.900	0.889	0.902

Table S6 – Individual data points (F/F_0) related to Fig. S5b

[PI(4)P]	F/F_0 (335 nm)									
	16:0/16:0-PI(4)P				18:1/18:1-PI(4)P			18:0/20:4-PI(4)P		
(μM)	n1	n2	n3	n4	n1	n2	n3	n1	n2	n3
0	0.778	0.780	0.766	0.782	0.773	0.761	0.790	0.793	0.788	0.785
0.25	0.828	0.830	0.816	0.840	0.916	0.900	0.921	0.934	0.957	0.958
0.5	0.876	0.882	0.865	0.907	0.976	0.954	0.976	0.994	1.002	0.996
0.75	0.913	0.915	0.896	0.960	0.987	0.963	1.002	1.003	1.010	0.999
1	0.937	0.933	0.914	0.973	1.003	0.963	1.031	1.010	1.012	1.005
1.25	0.948	0.940	0.933	1.001	1.014	0.965	1.020	1.017	1.011	1.020

Table S7 - Individual data points (fluo) related to Fig. S7d

	Fluorescence (a.u.)					
	$PI(4,5)P_2$			PI(4)P		
Total lipids (μM)	n1	n2	n3	n1	n2	n3
0	18027	15976	15408	15634	15024	13992
5	23345	19501	20825			
10	22805	23268	22142			
15	26347	24482	25256			
20	35246	27347	26090			
25	40763	29669	24861	18199	17684	16212
30	39599	28738	26675			
40	29972	30411	29666			
50	30431	31203	26948	18320	15777	16121
60	29796	31276	29289			
70	33880	31207	28592			
75				17590	16895	16493
80	32991	36286	30961			
90	31552	33376	29708			
100	33019	30373	31772	17691	18077	16448
125	36319	32019	32556	17017	19129	16526
150	36001	34892	33312	17437	17480	16366
175	37528	35223	31278	17402	16508	17603
200	37873	35580	32985	18237	17465	16878
225	37177	36105	31785			
250	35968	37192	33009	19588	18457	16664
275	37697	37451	31552			
300	37247	36062	31324	19070	18772	17500
350	36453	35773	32198			

Table S8 - Individual data points (Tm) related to Fig. S8e

	Tm (°C)		
Lipid subspecies	n1	n2	n3
16:0/16:0-PI(4)P	53.45	50.39	50.64
16:0/16:0-$PI(4,5)P_2$	44.64	44.85	
18:0/20:4-PI(4)P	61.72	62.55	
18:0/20:4-$PI(4,5)P_2$	46.29	44.68	45.06
DOPC only	45.73	43.19	43.17
